# Tissue origin and virus specificity shape human CD8^+^ T cell cytotoxicity

**DOI:** 10.1126/sciimmunol.adq4881

**Published:** 2025-07-18

**Authors:** Julia Niessl, Thomas R. Müller, Christian Constantz, Curtis Cai, Vera Nilsén, Olga Rivera Ballesteros, Sarah Adamo, Tobias Kammann, Elli Mouchtaridi, Yu Gao, Mily Akhirunnesa, Elisa J. M. Raineri, Whitney Weigel, Efthymia Kokkinou, Christopher Stamper, Anne Marchalot, John Bassett, Sabrina Ferreira, Inga Rodahl, Nicole Wild, Demi Brownlie, Emma Ringqvist, Malin Flodström-Tullberg, Sian Llewellyn-Lacey, Chris Tibbitt, Quirin Hammer, Jakob Michaëlsson, David A. Price, Jenny Mjösberg, Nicole Marquardt, Johan K. Sandberg, Takuya Sekine, Carl Jorns, Marcus Buggert

**Affiliations:** 1 Center for Infectious Medicine, Department of Medicine Huddinge, Karolinska Institutet, Stockholm, Sweden; 2 Center for Hematology and Regenerative Medicine, Department of Medicine Huddinge, Karolinska Institutet, Stockholm, Sweden; 3 Division of Infection and Immunity, Cardiff University School of Medicine, University Hospital of Wales, Cardiff, UK; 4 Systems Immunity Research Institute, Cardiff University School of Medicine, University Hospital of Wales, Cardiff, UK; 5 ME Transplantation, Karolinska University Hospital Huddinge, Stockholm, Sweden; 6 Department of Clinical Science, Intervention and Technology, Karolinska Institutet, Stockholm, Sweden

## Abstract

CD8^+^ T cells are classically defined by cytotoxic functionality. However, memory CD8^+^ T cells are extensively distributed across various niches in the human body, where these functions may be influenced by anatomical localization and antigen specificity. Here, we established a human organ donor cohort to investigate how CD8^+^ T cell cytotoxicity is regulated and shaped as a function of tissue location. Expression of the conventional cytotoxic molecules granulysin, perforin, and granzyme B was most prominent among peripheral blood CD8^+^ T cells and decreased progressively with residency status among lymphoid and nonlymphoid tissue CD8^+^ T cells, inversely mirroring the expression of CD69 and CD103. Other cytotoxic molecules, including granzymes A, H, K, and M, were variably expressed among memory CD8+ T cells across tissues in linkage with viral antigen specificity, with those targeting persistent viruses expressing multiple granzymes coordinately. Using an *in vitro* tonsil memory CD8^+^ T cell system, we found that TGF-β induced discordant expression of cytotoxic molecules and CD103, which could be partially overridden by extensive cell proliferation in the presence of IL-15. Comprehensive cytotoxic molecule CRISPR/Cas9 knock-out experiments further demonstrated that activated tonsil memory CD8^+^ T cells acquired redirected killing activity via mechanisms involving perforin and granzyme B. Our findings suggest that human memory CD8+ T cell cytotoxic activity is shaped by location and viral antigen specificity, and intricately regulated by environmental cues to fine-tune cytotoxic functions during both resting and activated states.

## Introduction

Direct killing of target cells is a fundamental feature of the anamnestic response of CD8^+^ T cells, vital for controlling malignancies and infections. Cytotoxic mechanisms are executed through the targeted delivery of cytolytic molecules, such as granzyme B, perforin, and granulysin, triggered by various inflammatory and antigenic stimuli. Such cytotoxic capabilities have predominantly been attributed to effector memory CD8^+^ T cells in humans (Buggert et al., 2023; Sallusto et al., 1999). However, the majority of studies examining cytotoxic functions in humans have focused on peripheral blood samples where more terminally differentiated effector memory cells are found, characterized by high expression levels of cytotoxic molecules (Buggert et al., 2020; Sallusto et al., 1999; Zwijnenburg et al., 2023). To prevent destructive tissue immunopathology, the cytotoxic apparatus is believed to be tightly regulated, exhibiting a reduced cytotoxic phenotype under steady-state conditions while maintaining the ability to rapidly upregulate its response to external stimuli upon re-infection (Beura et al., 2018; Rosato et al., 2023; Schenkel et al., 2013).

Tissue-resident memory (TRM) CD8^+^ T cells are pivotal in providing a first-line defense against secondary infections (Lange et al., 2022). TRM cells are characterized by a lack of recirculation during steady-state conditions and strategic compartmentalization at relevant anatomical sites across human lymphoid and nonlymphoid tissues, including tonsils, lungs, gut, liver, skin, spleen, as well as other organs, enabling them to confer rapid protection against infectious challenge (Christo et al., 2021; Miron et al., 2018). Studies of human TRM cells are expanding. Yet, these studies remain challenging to conduct given the difficulty of accessing tissues, particularly from the same donor, and by the inherent genetic diversity among humans, as well as the variety of infections – both acute and persistent – that individuals may encounter. Adding to this complexity, many tissues, such as the lung, liver, and spleen, are highly vascularized and therefore, “permissive,” accommodating both vascular and parenchymal memory CD8^+^ (mCD8^+^) T cell subsets (Kasmani et al., 2022). Currently, there is a significant gap in our understanding of how TRM cells function and target different viruses across nonlymphoid and lymphoid tissues, and how memory CD8^+^ T cells in these tissues are regulated by potential environmental cues. This underscores a critical need for further research to elucidate the mechanisms underlying TRM cell-mediated immunity and its regulation in response to diverse pathogenic challenges.

The human genome encodes, beyond the well-characterized serine protease granzyme B, an additional four members (granzyme A, H, K, and M), each possessing unique functional properties that contribute to cellular immunity (Chowdhury & Lieberman, 2008; Trapani, 2001). These granzymes are thought to play critical roles not only in the cytotoxic response of immune cells but also in modulating various other biological processes. Granzyme A, for instance, is a serine protease known for its cytotoxic and proinflammatory functions, enabling CD8^+^ T cells and natural killer (NK) cells to induce apoptosis in target cells. In addition, studies have demonstrated that granzyme A can activate key mediators of inflammatory pathways by proteolysis, such as procytokine IL-1β and gasdermin D, leading to the induction of proinflammatory responses and regulated cell death through pyroptosis (Zhou et al., 2020). Similarly, noncytotoxic functions have been reported also for the other members of the granzyme family (Anthony et al., 2010; Bouwman et al., 2021). These studies demonstrate the dual functionality of granzymes in both directly killing infected cells and modulating the immune environment to enhance the overall immune response. Despite these insights into the individual roles of granzymes, particularly from studies focusing on their expression in human blood, our understanding of their specific contributions to the killing capacity of human TRM cells remains incomplete. While recent advancements in tissue-specific immune profiling have offered unprecedented views of the immune landscape across different human tissues (Consortium et al., 2022; Madissoon et al., 2022; Massoni-Badosa et al., 2022), these studies often do not elucidate the functional significance and expression patterns of these molecules in tissue-resident immune surveillance. This underscores the need for further research to dissect the expression patterns of each cytotoxic molecule across the human body, their linkages to residency and viral specificity, and to understand whether they are involved in killing mechanisms.

In this study, we analyzed cytotoxic CD8^+^ T cell functions across tissues in a human organ donor cohort, revealing that the expression of classical cytotoxic molecules is highest in peripheral blood and inversely correlates with the CD8^+^ TRM status in multiple tissues. We observed a notable heterogeneity in the expression of most granzymes, influenced by the type of viral antigens CD8^+^ T cells target. Additionally, our data showed that different microenvironmental cytokines can differentially alter CD8^+^ TRM formation and cytotoxicity, indicating that CD8^+^ TRM cytotoxic activity is finely tuned by location, viral antigen history, and environmental cues to enable diverse effector functions.

## Results

### The conventional cytotoxic phenotype of memory CD8^+^ T cells is predominantly observed in peripheral blood

**Fig 1:**
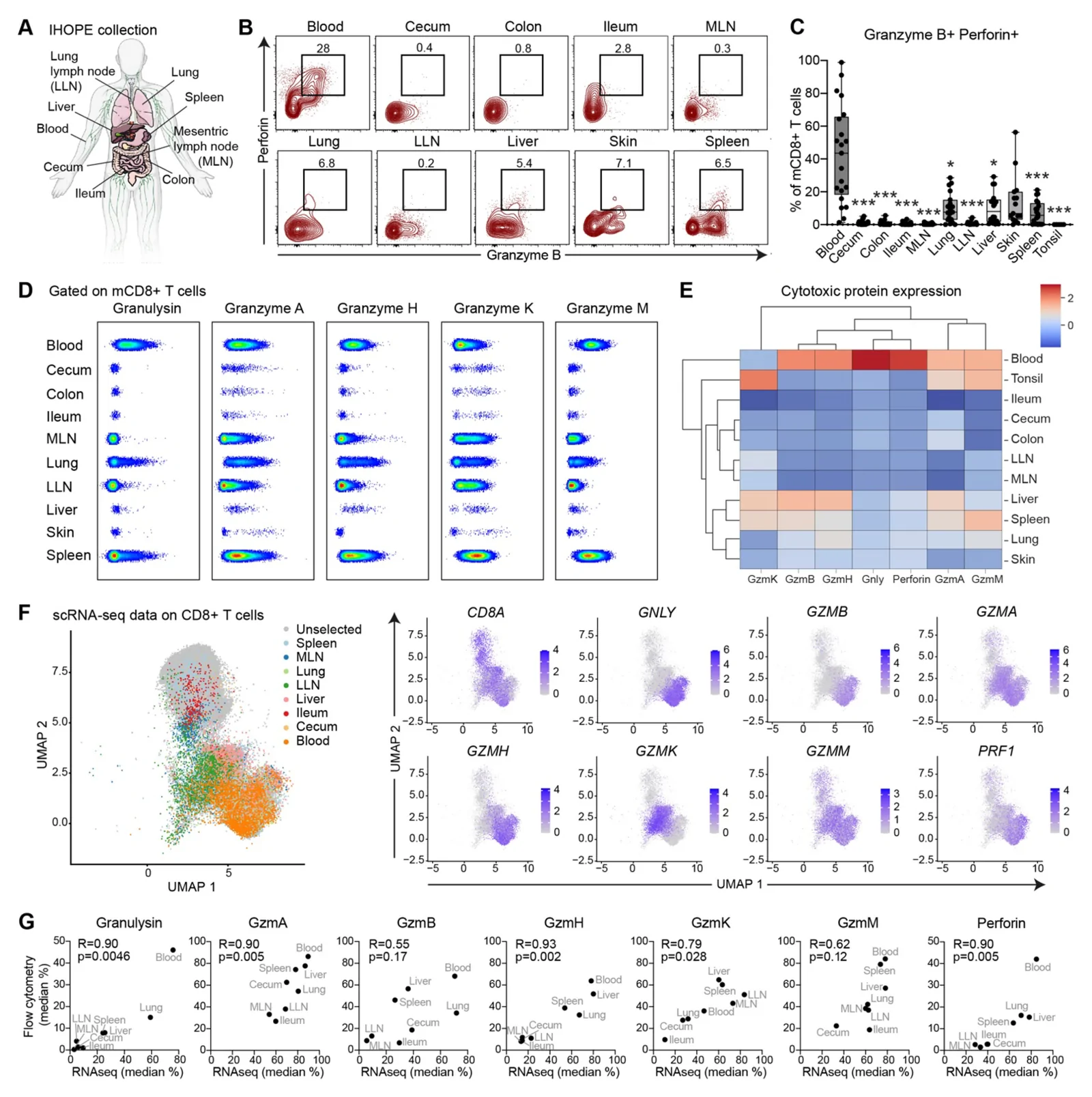
Cytotoxic molecule expression in mCD8^+^ T cells across human tissues. **(A)** Illustration of tissues/organs collected in the IHOPE cohort. **(B)** Representative FACS plot showing granzyme B and perforin expression in mCD8^+^ T cells across all tissues from one IHOPE donor. **(C)** Cumulative data on the frequency of granzyme B/perforin double-positive mCD8^+^ T cells in all assessed human tissues. Kruskal-Wallis test with Dunn’s post-test. **(D)** Representative expression of granulysin and other granzyme family members (A, H, K, M) in mCD8^+^ T cells across all tissues from one IHOPE donor. **(E)** Hierarchical clustering of median expression levels (z-score) of the different granzymes (Gzm), granulysin (Gnly), and perforin in mCD8^+^ T cells in all tissues (based on 19-41 donors depending on the tissue). **(F)** Single-cell RNA sequencing (scRNA-seq) data (Domínguez Conde et al., 2022) of mCD8^+^ T cells. Annotated and manually curated by Celltypist, followed by cytotoxic molecule expression analysis across tissues. **(G)** Correlative analysis (Spearman) depicting the relationship between in-house flow cytometry-derived protein data and corresponding mRNA transcript data curated from the publicly available dataset. *P < 0.05, **P < 0.01, ***P < 0.001.

Classically, cytotoxic functions of CD8^+^ T cells have been primarily associated with the expression of granzyme B (GzmB) and perforin. Previous research, involving both mice and humans, has shown that this conventional cytotoxic phenotype of CD8^+^ T cells is predominantly observed in the blood and, to a lesser extent, in recirculating CD8^+^ T cells, as evidenced through studies of efferent lymphatics (Buggert et al., 2020; Gerlach et al., 2016), whereas certain cytolytic effector proteins are clearly less expressed in human gastrointestinal mucosa (Kiniry et al., 2018; Shacklett et al., 2004). To thoroughly investigate cytotoxic properties, we utilized matched tissue samples from a human organ donor cohort established at Karolinska Institutet, known as the Immunology Human Organ Donor Programme (IHOPE cohort, n=25) (Table S1). These samples encompassed peripheral blood, spleen, liver, ileum, cecum, colon, mesenteric lymph nodes (MLNs), lung, lung lymph nodes (LLNs), and skin (Fig. 1A). Additionally, tonsil and matched blood samples were obtained from a second cohort, the TONCIM cohort (n=21) (Fig. S1A-C, Table S1).

We first investigated mCD8^+^ T cells (Fig. S2A) coexpressing perforin and granzyme B (GzmB) (Fig. 1B) and discovered significantly lower frequencies across all assessed tissues compared to blood (Fig. 1B-C). Additionally, we assessed other serine proteases and pore-forming molecules – granzyme A (GzmA), granzyme H (GzmH), granzyme M (GzmM), granzyme K (GzmK), and granulysin – across all tissue sites (Fig. 1D), and found that a majority of these proteins exhibited their highest expression in blood compared to most other tissue compartments (Fig. 1D-E). Notably, GzmK expression was highest in tonsillar mCD8^+^ T cells (Fig. 1E), indicating a distinct expression pattern of this specific serine protease by lymphoid mCD8^+^ T cells.

To ascertain whether the distinct expression patterns of the investigated cytotoxic molecules are also evident at the RNA level, we utilized a publicly available cross-tissue immune cell atlas, which contains single-cell RNA sequencing (scRNA-seq) data for over 300,000 immune cells from 16 tissue sites collected from 12 deceased human organ donors (Domínguez Conde et al., 2022). We selected matched tissues corresponding to samples obtained from our cohorts. Memory CD8^+^ T cells, annotated and manually curated by Celltypist, were analyzed for their expression of GzmA, GzmB, GzmH, GzmK, GzmM, perforin and granulysin (Fig. 1F). In line with the findings at the protein level, scRNA-seq data unveiled tissue compartment-dependent differential expression of cytotoxic molecules (Fig. 1F). Blood and vascularized organs, such as the spleen, liver, and lung, showed a higher frequency of mCD8^+^ T cells with detectable mRNA levels of *GZMA, GZMB, GZMH, GZMM, PRF*, and *GNLY*, whereas mRNA levels of *GZMK* were elevated in mCD8^+^ T cells in lymph nodes (Fig. 1F, Fig. S2B). The median frequencies of mCD8^+^ T cells expressing cytolytic markers in the tissue immune atlas scRNA-seq dataset positively correlated with frequencies obtained by protein staining in our independent IHOPE cohort (Fig. 1F). Collectively, these findings indicate that, contrary to peripheral blood, a significant proportion of lymphoid and nonlymphoid tissue mCD8^+^ T cells display markedly lower expression levels of many serine proteases and pore-forming molecules at steady-state.

### Early-differentiated and tissue-resident mCD8^+^ T cells display limited expression of cytotoxic molecules in most tissues

**Fig. 2:**
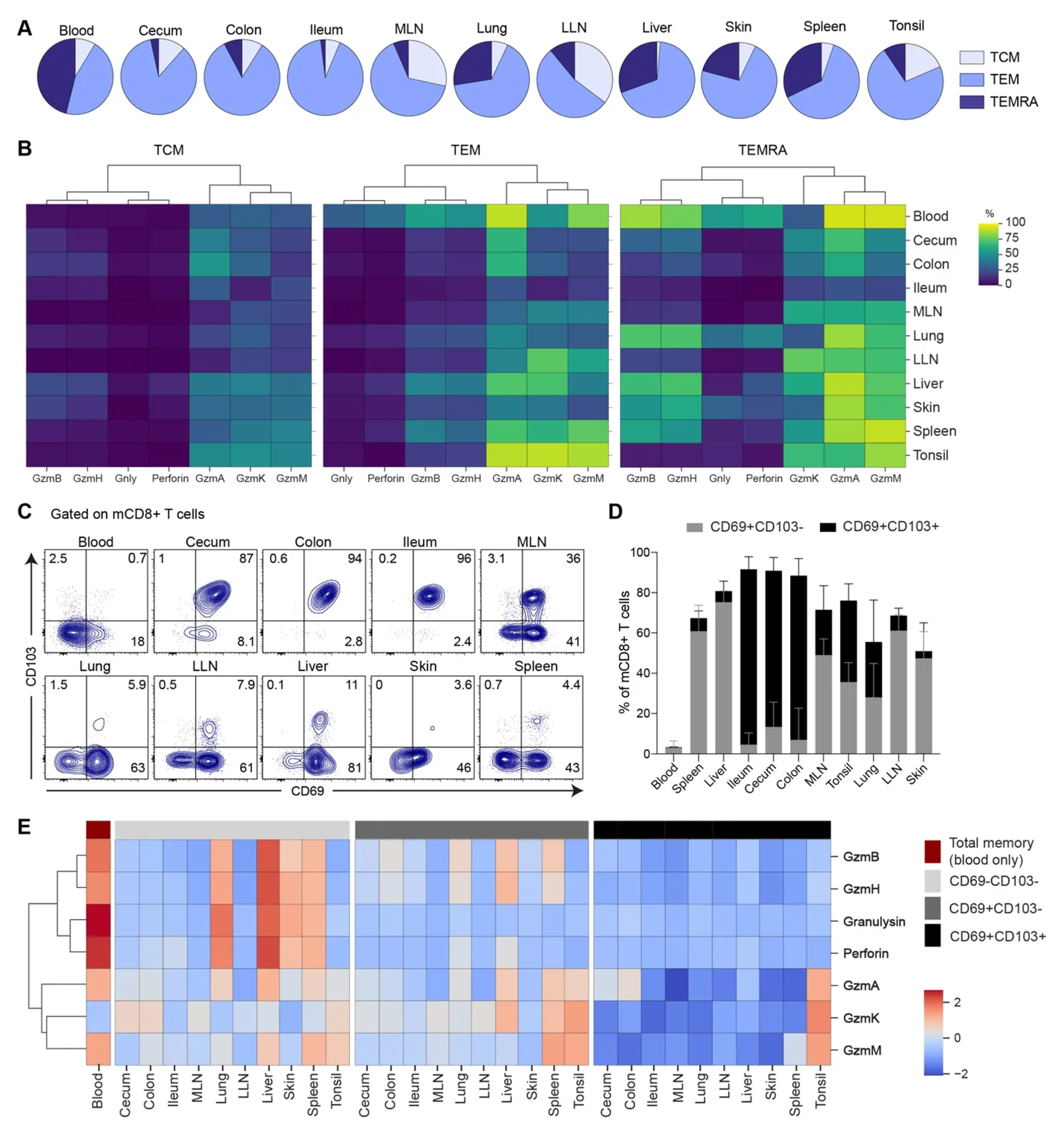
Expression of cytotoxic molecules in tissues is associated with the memory phenotype and residency status of mCD8^+^ T cells. **(A)** Pie chart illustrating the relative abundance of CCR7^+^CD45RA^-^ (central memory; TCM), CCR7^-^CD45RA^-^ (effector memory; TEM), and CCR7^-^ CD45RA^+^ (effector memory RA+; TEMRA) subsets of CD8^+^ T cells across tissues (based on 16-40 donors depending on the tissue). **(B)** Heatmap showing the median expression of cytotoxic molecules by flow cytometry within the CD8^+^ TCM (left), TEM (middle), and TEMRA (right) T cell subsets across all tissues (based on 16-40 donors depending on the tissue). **(C)** Representative flow cytometry plots of tissue residency markers CD69 and CD103 in mCD8^+^ T cells from one IHOPE donor, and **(D)** cumulative data on the frequency of CD69 and CD103 coexpressing mCD8^+^ T cells in human tissues (based on 16-40 donors depending on the tissue **(E)** Heatmap displaying the expression of cytotoxic molecules (z-score) by mCD8^+^ T cells across tissues, categorized by CD69 and CD103 coexpression patterns (based on 16-40 donors depending on the tissue).

Peripheral blood mCD8^+^ T cells exhibited the highest expression of most cytolytic markers compared to their tissue-resident counterparts. However, organs such as the lung, spleen, and liver are highly perfused and likely contain a substantial fraction of vascular mCD8^+^ T cells with an effector memory phenotype. Upon analyzing the expression of cytotoxic molecules across central memory (TCM), effector memory (TEM), and effector memory RA^+^ (TEMRA) CD8^+^ T cell subsets across all tissues (Fig. 2A), we observed a generally increased expression of most cytotoxic molecules in TEMRA cells, especially in vascularized organs (Fig. 2B). Notably, there was significant heterogeneity in the expression of cytotoxic molecules, particularly within the TEM subset, which may reflect additional factors such as the tissue-residency status of mCD8^+^ T cells (Fig. 2B). We extended our analysis to mCD4^+^ T cells (Fig. S3A), which are typically considered less cytotoxic, and observed a skewed expression of most cytotoxic molecules in both circulation and vascularized organs, predominantly in the liver (Fig. S3B-C). These data indicate that cytotoxic molecule expression extends to other lymphocyte subsets in the blood, suggestive of a broader cytotoxic immune imprint in the blood that extends beyond classical cytotoxic T cells.

To distinguish tissue-resident from potentially circulating mCD8^+^ T cells in different tissues, we next identified distinct mCD8^+^ T cell subsets based on their expression of tissue-resident markers CD69 and CD103 (Fig. 2C-D). The majority of mCD8^+^ T cells expressed CD69 in all examined tissues, whereas the frequency of CD103^+^ TRM cells varied across different compartments (Fig. 2D). As previously reported (Kumar et al., 2017), CD69^+^CD103^+^ TRM cells were predominant in the intestinal and respiratory tracts, whereas CD69 single-positive cells were more common in lymph nodes, spleen, liver, and skin (Fig. 2D). We then compared the cytotoxic profile of CD69^+^CD103^+^, CD69^+^CD103^-^, and CD69^-^CD103^-^ mCD8^+^ T cells in tissues relative to total peripheral blood mCD8^+^ T cells. Unsupervised analysis revealed that GzmB, GzmH, granulysin, and perforin formed one cluster, with GzmA, GzmM, and GzmK forming another (Fig. 2E). CD69^-^CD103^-^ T cells present in the liver, spleen, lung, and skin more closely resembled blood mCD8^+^ T cells and were characterized by the highest expression levels of most cytotoxic molecules, except for GzmK (Fig. 2E). CD69^+^CD103^-^ mCD8^+^ T cells displayed intermediate expression of many cytotoxic molecules, with particularly high expression levels of GzmA, GzmK, and GzmM observed in tonsil and spleen mCD8^+^ T cells (Fig. 2E). Notably, the bona fide TRM population, CD69^+^CD103^+^ T cells, showed the lowest frequency of all investigated cytotoxic molecules from most tissues, except for tonsils showing high expression levels of GzmA, GzmK, and GzmM (Fig. 2E). Together, our findings demonstrate that the levels of cytotoxic markers in various organs are biased by the presence of circulating effector memory T cells, and that low levels of all serine proteases and pore-forming molecules are characteristic of tissue-resident CD103^+^ mCD8^+^ T cells.

### Cytotoxic mCD8^+^ T cell capacity is influenced by virus specificity and tissue origin

**Fig. 3:**
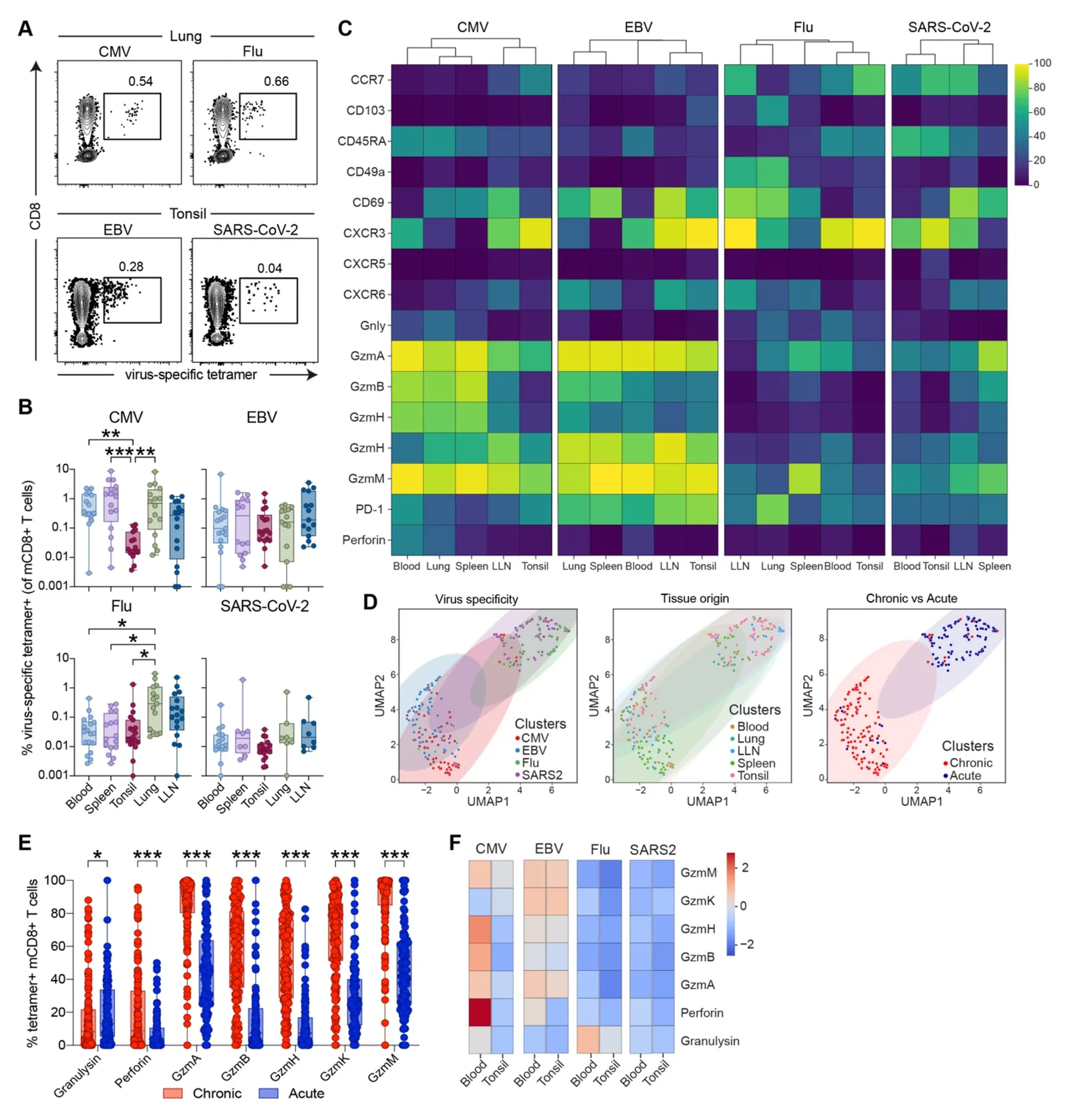
The cytotoxic potential of mCD8^+^ T cells is influenced by virus persistency and tissue origin. **(A)** Representative flow cytometry plots of MHC-Class I tetramer staining for identifying CMV-, EBV-, Flu-, and SARS-CoV-2-specific mCD8^+^ T cells in tissues. **(B)** Relative abundance of virus-specific mCD8^+^ T cells in the assessed human tissues. **(C)** Heatmap illustrating the median expression of cytotoxic, tissue retention, and differentiation markers in different virus-specific T cell subsets (based on 12-16 donors for CMV, 8-18 donors for EBV, 9-16 donors for Flu, 5-18 donors for SARS-CoV-2 depending on the tissue. Analysis of SARS-CoV-2 specific mCD8^+^ T cells in the lung was not conducted due to limited cell number. **(D)** UMAP projection of combined expression patterns based on flow cytometry data, colored based on the following parameters: virus specificity, tissue origin, and whether the virus infection is persistent or acute. Each dot represents the cumulative data for each virus-specific mCD8^+^ T cell population from a given tissue. **(E)** Comparison of cytotoxic molecule expression in virus-specific mCD8^+^ T cells between persistent (CMV + EBV) and acute (Flu + SARS-CoV-2) infections. **(F)** Heatmap displaying the median expression levels (z-score) of various cytotoxic molecules in virus-specific mCD8^+^ T cells between tonsils and blood. Analysis was done using Kruskal-Wallis test with Dunn’s post test (B) or Mann-Whitney test (E) with *P < 0.05, **P < 0.01, ***P < 0.001.

Although CD8^+^ TRM cells clearly correlated with reduced potential cytotoxic potential, we observed significant heterogeneity in the expression of cytotoxic molecules among mCD8^+^ T cells both between individuals and across tissues. Previous studies have established that cytotoxic functions can vary among mCD8^+^ T cells with different specificities (Harari et al., 2009). For example, mCD8^+^ T cells specific to cytomegalovirus (CMV) are known for their exceptionally high cytotoxic activity (Appay et al., 2000; Klenerman & Oxenius, 2016). Additionally, CMV-specific T cells are predominantly found in peripheral blood and may, therefore, impact our observations regarding the differences between blood and tissue compartments. To investigate this further, we analyzed mCD8^+^ T cell responses specific to CMV, Epstein-Barr virus (EBV), influenza virus (Flu), and SARS-CoV-2 in peripheral blood, spleen, and tissues linked to both upper and lower respiratory tract (tonsils, lung, LLNs) using MHC class I tetramer staining (Fig. 3A). Consistent with prior findings (Niessl et al., 2021), CMV-specific mCD8^+^ T cell frequency was highest in peripheral blood, spleen, and lung, and was scarcely detectable in tonsils (Fig. 3B). EBV-specific mCD8^+^ T cells were similarly distributed across all examined organs but tended to be more abundant in tonsils compared to other virus-specificities (Fig. 3B). Flu-specific mCD8^+^ T cells were primarily located in the lungs and LLNs, whereas SARS-CoV-2-specific mCD8^+^ T cells were found at similar low frequencies across all tissues, with a tendency for higher frequencies in the lungs and LLNs (Fig. 3B).

Further investigation into the phenotype and function of these virus-specific mCD8^+^ T cell populations revealed that characteristics varied depending on both the tissue origin and the specificity of the cells (Fig. 3C, Fig. S4A). For instance, TRM status was generally low in all tissues for CMV and SARS-CoV-2-specific mCD8^+^ T cells (Fig. 3C, Fig. S3B). In contrast, EBV-specific mCD8^+^ T cells exhibited a high frequency of CD69 and CD103 coexpression across several tissues and, notably, increased coexpression of CD69 and CD49a in the tonsils, the primary site of infection, compared to the spleen, lung, and LLNs. Conversely, Flu-specific mCD8^+^ T cells displayed significantly higher expression of the TRM markers CD103, CD49a, CXCR6, and CXCR3 in the lung and LLNs, indicating a distinct residency status of virus-specific mCD8^+^ T cells determined by the primary site of infection (Fig. 3C, Fig. S4B).

We next examined the cytotoxic profile of all virus-specific mCD8^+^ T cell subsets. Uniform manifold approximation and projection (UMAP) analysis identified distinct profiles based on viral specificity rather than tissue origin (Fig. 3D). Clustering was most pronounced when comparing mCD8^+^ T cells with persistent (CMV and EBV) versus acute (Flu and SARS-CoV-2) viral specificities, with persistent virus-specific mCD8^+^ T cells showing significantly higher frequencies of most assessed cytotoxic molecules (Fig. 3E). Notably, each specificity was correlated with a unique cytotoxic mCD8^+^ T cell profile (Fig. 3D-F). Although persistent virus-specificities generally demonstrated elevated levels of granzymes, normalization for each specificity revealed distinct profiles depending on the tissue origin of the virus-specific mCD8^+^ T cells (Fig. S4C), with a higher expression of cytotoxic molecules in blood and vascularized organs compared to lymphoid tissues (Fig. 3F, Fig. S4C). Particularly, CMV-specific CD8^+^ T cells showed lower expression levels of various granzymes in tonsils compared to blood, and most virus-specific CD8^+^ T cell populations exhibited reduced expression of the pore-forming molecules in tonsils compared to blood. (Fig. 3F). In summary, these findings illustrate that both the nature of the viral infection and the tissue location significantly influence the expression of the cytotoxic molecule repertoire in mCD8^+^ T cells.

### Distinct functional profiles between blood and tonsil mCD8^+^ T cells

**Fig. 4:**
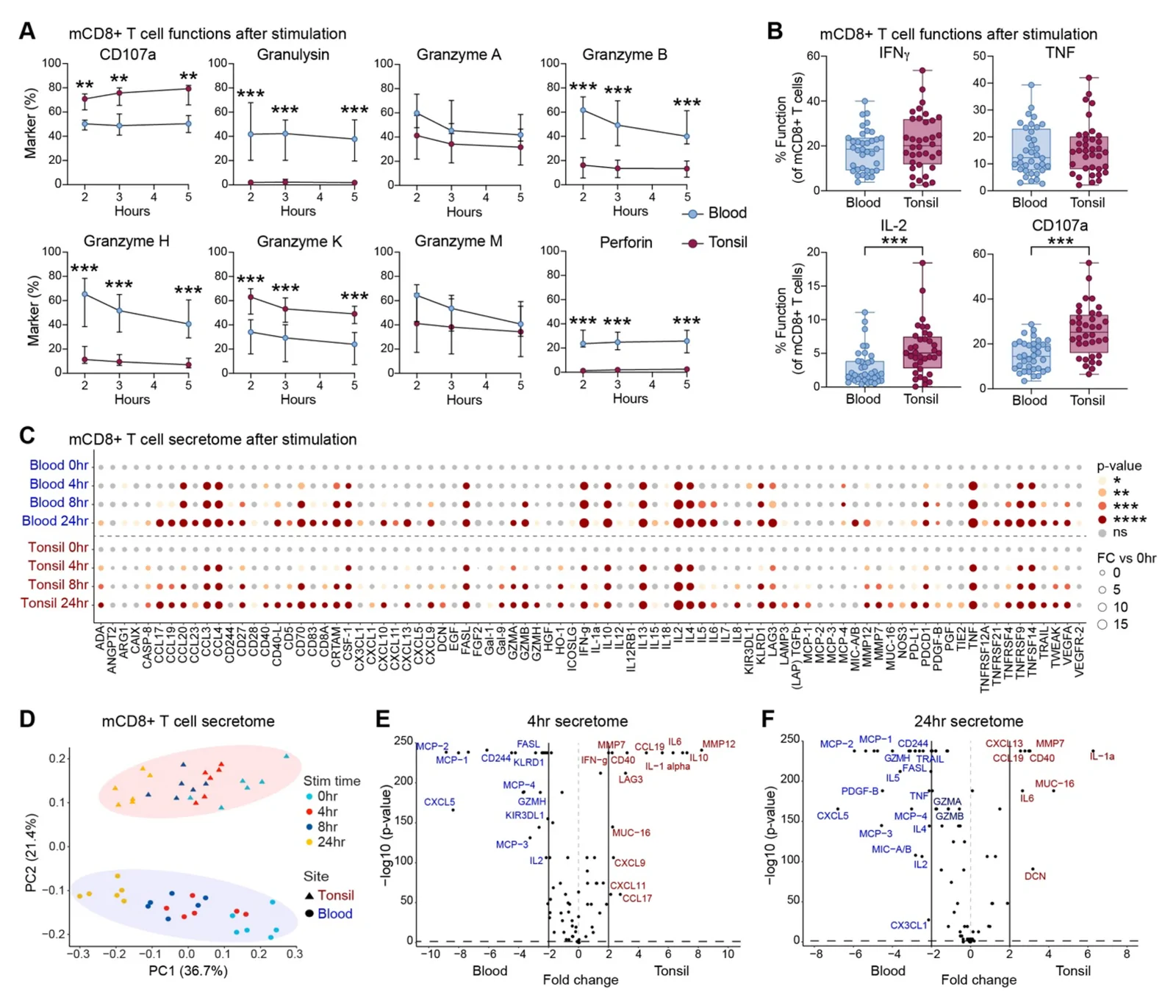
Kinetic and functional differences between mCD8^+^ T cells in blood and tissue. **(A)** Comparative analysis of cytotoxic molecule expression between blood and tonsil mCD8^+^ T cells over the time courses of 2 hours, 3 hours, and 5 hours post-SEB stimulation (10 donors for blood, 9 donors for tonsil group). **(B)** Comparative analysis of IFNγ, TNF, IL-2, and CD107a expression between blood and tonsil SEB-specific mCD8^+^ T cells following 12-hour stimulation. **(C)** Secretome analysis of blood and tonsil mCD8^+^ T cells at various time points after TCR (anti-CD3/CD28/CD2) stimulation (5 donors per group). The size of the bubbles represents the magnitude of protein secretion (fold change over the unstimulated control at 0 hours). The color code represents p-values compared to the 0-hour time point. **(D)** Principal component analysis (PCA) comparing all variables in the secretome dataset of blood and tonsil mCD8^+^ T cells over the time course of 0 hours, 4 hours, 8 hours, and 24 hours post-TCR stimulation. Volcano plots illustrate differentially secreted soluble factors between blood and tonsil mCD8^+^ T cells at **(E)** 4 hours and **(F)** 24 hours, respectively. Analysis was done using Mann-Whitney test with correction for multiple comparisons using Holm-Šídák (A) or Mann-Whitney test (B). *P < 0.05, **P < 0.01, ***P < 0.001.

We next sought to examine the functional kinetics of mCD8^+^ T cells in blood and tissue. For this purpose, mCD8^+^ T cells from blood and tonsils were subjected to short-term TCR stimulation for 2, 3, and 5 hours using staphylococcal enterotoxin B (SEB). Notably, tonsil mCD8^+^ T cells rapidly upregulated CD107a already two hours after stimulation, indicating robust degranulation early after stimulation despite low expression and induction of multiple cytotoxic molecules (Fig. 4A). We further observed that mCD8^+^ T cells from blood exhibited sustained elevated expression of most cytotoxic molecules compared to tonsil mCD8^+^ T cells after stimulation (Fig. 4A). To determine the ability of tissue-derived mCD8^+^ T cells to respond promptly to TCR stimulation we evaluated additional functional parameters, which revealed comparable levels of IFN-γ and TNF, whereas IL-2 and the degranulation marker CD107a were more highly expressed in mCD8^+^ T cells from tonsils compared to those from blood (Fig. 4B).

To extend these analyses, we stimulated CD45RA-mCD8^+^ T cells from tonsils and blood with anti-CD3/CD28 for durations of 0, 4, 8, and 24 hours, followed by multiparametric proximity extension assay (Olink) of the supernatants to assess 92 functional parameters. As expected, blood and tonsil mCD8^+^ T cells showed increased production of multiple cytokines, chemokines, and other molecules over time, compared to the initial measurement at 0 hours. (Fig. 4C). Notably, mCD8^+^ T cells from tonsils and blood exhibited markedly different secretome profiles both early and late after TCR stimulation (Fig. 4D, Fig. S5). Shortly after stimulation (4 hours), multiple cytokines (IFN-γ, IL-10, IL-6, IL-1α), chemokines (CCL17, CCL19, CXCL9, CXCL11, CXCL13), activation molecules (TNFRSF4, CD40), and matrix metalloproteinases (MMP7, MMP12) were predominantly secreted by tonsil mCD8^+^ T cells (Fig. 4E, Fig. S5). In contrast, cytotoxic and late differentiation molecules (FASL, GZMH, KLRD1, CD244), as well as monocyte chemoattractant proteins (MCP-1, MCP-2, MCP-3, MCP-4), were more abundantly produced by blood mCD8^+^ T cells 4 hours after stimulation (Fig. 4E, Fig. S5). At a later time point (24 hours post-stimulation), blood mCD8^+^ T cells increased their release of a wide array of cytotoxic molecules (FASL, GZMH, GZMA, GZMB, TRAIL), cytokines (TNF, IL-2, IL-5, IL-4), and chemokines (CXCL1, CXCL5, MCP-1, MCP-2, MCP-3, MCP-4), while a specific set of chemokines (CXCL13, CCL19), cytokines (IL-6, IL-1α) and matrix metalloproteinases (MMP7, MMP12) were still elevated in tonsil mCD8^+^ T cells (Fig. 4F, Fig. S5). These analyses suggest that mCD8^+^ T cells in tonsils can rapidly upregulate a distinct set of functions, whereas over time, blood mCD8^+^ T cells demonstrate a more comprehensive functional profile, including the upregulation of numerous cytotoxic molecules and cytokines critical for host responses.

### Cytotoxic functions of tonsil mCD8^+^ T cells are differentially regulated by IL-15 and TGF-β

**Fig. 5:**
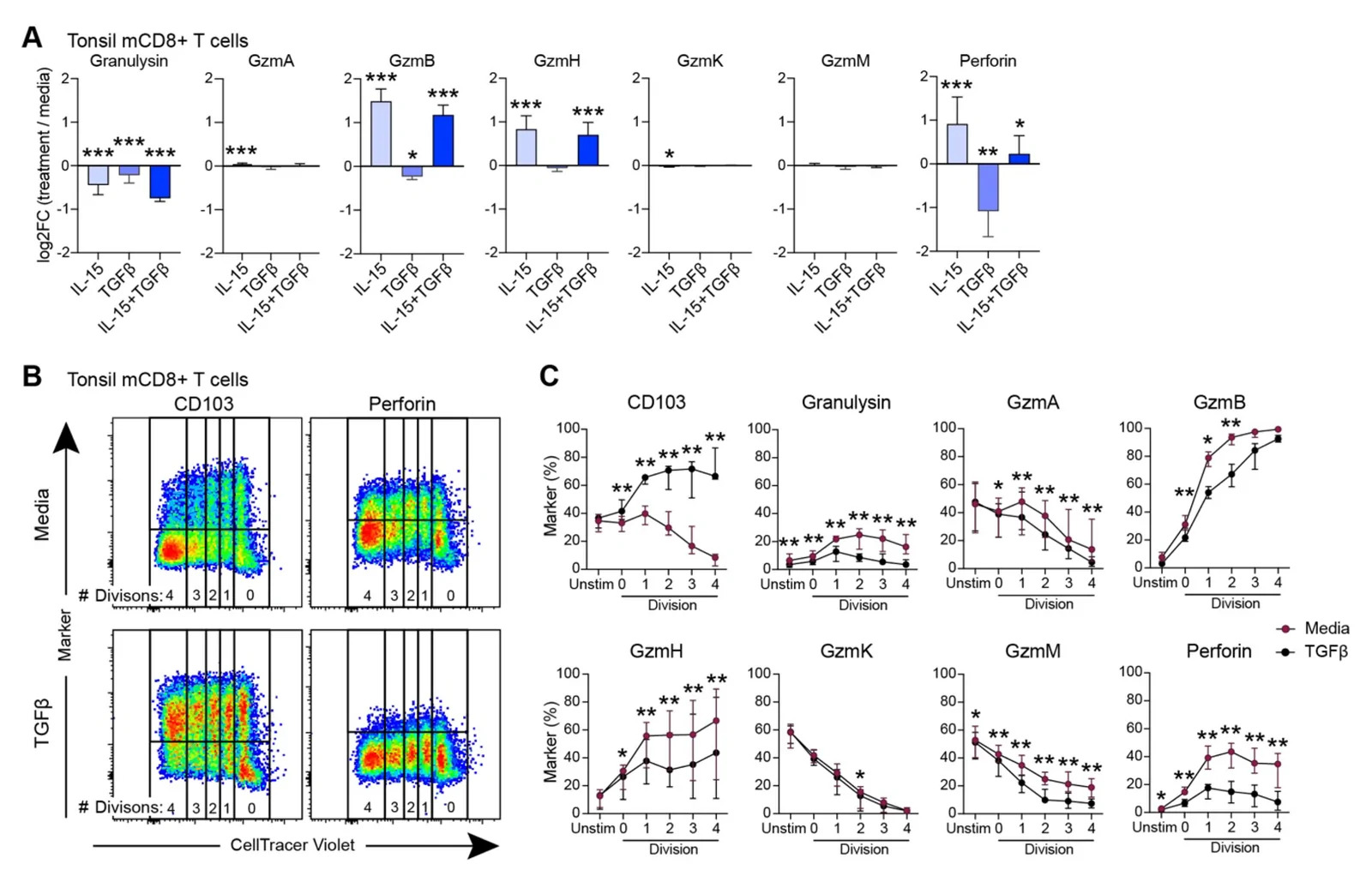
Regulation of cytotoxic molecule expression in mCD8^+^ T cells is dynamically influenced by IL-15 and TGF-β. **(A)** Relative changes of cytotoxic molecule expression in tonsil mCD8^+^ T cells responding to IL-15, TGF-β, or both combined. Data are displayed as fold change over the untreated control after 18 hours of stimulation. Median ± IQR (12 donors per group). **(B)** Representative flow cytometry plots show the effects of mCD8^+^ T cell division following SEB stimulation on tissue residency (CD103 expression) and cytotoxicity (perforin), influenced by the presence of TGF-β. **(C)** Aggregated comparative analysis illustrating the relationship between mCD8^+^ T cell division and cytotoxic molecule expression during the absence or presence of TGF-β. Median ± IQR (11 donors per group). Analysis was done using Wilcoxon matched-pairs signed rank test (A) or with correction for multiple comparisons using Holm-Šídák (C). *P < 0.05, **P < 0.01, ***P < 0.001.

The observation of diminished expression of cytotoxic molecules in tonsil mCD8^+^ T cells shortly after TCR stimulation prompted us to consider whether other factors might also influence cytotoxicity. Beyond TCR signaling, the development and maintenance of TRM cells are influenced by both soluble and nonsoluble factors, including IL-15 and TGF-β (Mackay et al., 2015). Given that both cytokines may affect the expression of cytotoxic proteins in immune cells (Jabri & Abadie, 2015; Taber et al., 2023), we explored their impact on tissue mCD8^+^ T cells. Unlike stimulation with SEB, short-term exposure of tonsil mCD8^+^ T cells to IL-15 or TGF-β, without concurrent TCR stimulation, resulted in significant changes in the expression of specific cytotoxic molecules. IL-15 markedly increased the expression of perforin, GzmB, and GzmH compared to media control, while exerting minimal effects on GzmA, GzmK, and GzmM (Fig. 5A). Conversely, TGF-β treatment significantly decreased the expression of perforin as well as GzmB and suppressed the induction of these molecules by IL-15 when both were applied together (Fig. 5A).

We further investigated whether the suppressive effect of TGF-β on the induction of cytotoxic molecules could be observed during active cell proliferation. Stimulation of tonsil mCD8^+^ T cells with SEB for four days led to an increase in perforin, GzmB, and GzmH expression levels, whereas the expression of GzmA, GzmK, and GzmM progressively decreased over time (Fig. 5B-C). CD103 expression, a surrogate marker for cellular residency, showed a progressive decline in tonsil mCD8^+^ T cells over time (Fig. 5B). As expected, CD103 expression progressively increased over the assay period in the presence of TGF-β (Fig. 5C). In contrast, in the presence of TGF-β, the expression levels of all cytolytic molecules were significantly reduced throughout the assay period, with the exception of GzmK (Fig. 5C). Proliferating mCD8^+^ T cells exposed to TGF-β did not reach the cytotoxic protein expression levels observed under conditions without TGF-β at any point during the four days of evaluation (Fig. 5C). These findings collectively suggest that the cytotoxic profiles of TRM cells are differentially regulated and fine-tuned by the presence of IL-15 and TGF-β.

### Differential roles of cytotoxic proteins in tonsil mCD8^+^ T cell-mediated killing

**Fig. 6:**
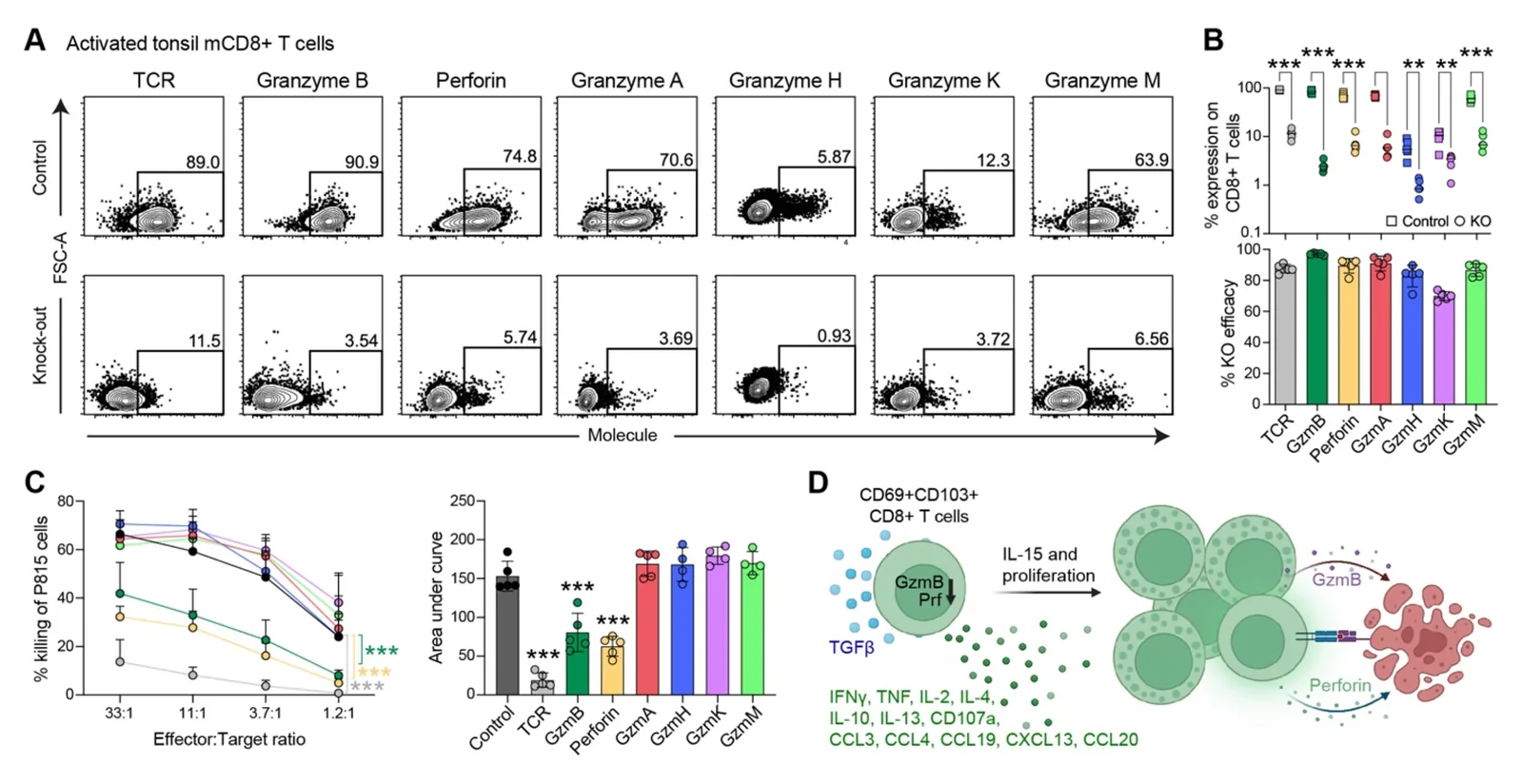
Primed tonsil mCD8^+^ T cells rely on perforin and granzyme B for effective redirected killing. **(A)** Representative flow cytometry plots demonstrating the efficacy of CRISPR knockout (KO) experiments targeting multiple cytotoxic molecules in activated (anti-CD3/CD28/CD2 supplemented with IL-2, IL-7, and IL-15) tonsil mCD8^+^ T cells. The TCRαβ was also knocked out as a control, with CD3 stainings used as a proxy to assess the efficacy of the KO. **(B)** Collective expression levels of KO targets (top) and overall % KO efficiency achieved (bottom) (4 donors per group). **(C)** Cytotoxic activity of mCD8^+^ T cells isolated post-KO of cytotoxic molecules. Redirected killing was quantified at different effector-to-target ratios against sensitized mastocytoma cells (P815) (left), and area under the curve for killing capacity influenced by various KO targets (right). **(D)** Illustration of early cytokine and chemokine expression patterns as well as subsequent functional killing mechanisms, guided by specific environmental signals. *P < 0.05, **P < 0.01, ***P < 0.001.

Finally, we sought to determine the functional significance of the distinct expression patterns of cytotoxic proteins observed in human tissue mCD8^+^ T cells. To elucidate the roles of cytotoxic molecules in mediating cell killing, we specifically targeted GzmA, GzmB, GzmH, GzmK, GzmM, and perforin using CRISPR/Cas9-mediated knockout in tonsil mCD8^+^ T cells activated by TCR and IL-2/-7/-15 stimulation (Fig. 6A). Subsequently, we assessed mCD8^+^ T cells’ killing capacity through a P815 redirected killing assay. Additionally, a complete TCR αβ knockout (KO) served as a null control to evaluate TCR-mediated target cell killing (Fig. 6A). We achieved a high efficiency of KO across all target molecules examined (Fig. 6A-B). When challenged with CD3-coated P815 target cells, both perforin-KO and GzmB-KO tonsil mCD8^+^ T cells exhibited a significant decrease in their cytotoxic capacity compared to the unmodified controls (Fig. 6C). This reduction was marginally more pronounced in perforin-KO cells than in GzmB-KO cells (Fig. 6C). Notably, very limited killing activity was observed in TCR αβ KO cells, attributable to the absence of surface CD3, which precludes TCR engagement (Fig. 6C). Contrary to our expectations, the KO of GzmA, GzmH, GzmK, and GzmM in mCD8^+^ T cells had minimal impact on their ability to kill target cells (Fig. 6C). In conclusion, these findings suggest that cytotoxicity in primed mCD8^+^ T cells from tissues is predominantly mediated by the cytotoxic molecules, perforin and GzmB (Fig. 6D), while other granzymes, such as GzmA, GzmH, GzmK, and GzmM, appear to play a minimal role in the killing capacity of tissue mCD8^+^ T cells.

## Discussion

TRM cells are strategically positioned throughout the human body to provide local immune surveillance. These cells are tailored to muster potent recall responses, and their successful establishment and compartmentalization in tissues are crucial for ensuring efficient protection against secondary viral challenges. However, most studies that have defined TRM functions are based on mouse models, and given the complexity of human biology, it is reasonable to believe that human TRM cells might function and adapt to their environment differently. This complexity underscores the potential for distinct cellular behaviors in humans, possibly diverging from those observed in model organisms. In our study, utilizing a human organ donor cohort, we explored the regulation and function of cytotoxic CD8^+^ T cells across tissues. We revealed that the expression of cytotoxic molecules is highest in peripheral blood and inversely related to the resident-memory status in various tissues. Cytotoxic molecule expression was significantly influenced by the tissue of origin and the nature of the virus targeted. Moreover, we discovered that the cytotoxic activity of tonsil memory CD8^+^ T cells is intricately regulated by environmental factors such as TGF-β and IL-15, which significantly impact their effector functions and their ability to adapt to resting and activated states.

Comprehensive studies have started to elucidate the tissue-specific prerequisites for TRM formation and maintenance, revealing that different bodily locations harbor unique memory phenotypes and differentiation states (Kumar et al., 2017; Miron et al., 2018; Thome et al., 2014). Despite this progress, the molecular mechanisms determining the cytolytic potential of TRM cells in tissues remain largely elusive. Traditionally, serine proteases and pore-forming proteins have been indicative of various cell development stages in cytotoxic lymphocytes and served as markers of memory differentiation (Bengsch et al., 2018; Bratke et al., 2005). However, identifying the factors that define the cytolytic profiles of virus-specific TRMs in human tissues has been challenging due to the difficulty in obtaining human tissue samples from the same donor. By analyzing human organ donors with paired tissue samples, we confirmed the strong association of serine proteases and pore-forming units with more differentiated mCD8^+^ T cells in the blood. Conversely, most tissues showed an inverse relationship between cytotoxic molecule expression and TRM status. Employing MHC-class I tetramers to identify T cells against specific viruses (CMV, EBV, Flu, and SARS-CoV-2), our findings suggest that the cytotoxic profile of TRM cells is influenced by both tissue origin and virus specificity. Indeed, the identity of tissue-resident lymphocytes is influenced by multiple factors, including gender, age, tissue ontogeny, and specificity (Brenes et al., 2021; Poon et al., 2021). Our comprehensive *ex vivo* analysis, involving a larger human cohort and additional virus specificities, further clarifies the compartmentalization of virus-specific memory T cells across tissues and sheds light on how cytolytic molecules, including the full granzyme spectrum and pore-forming units, are acquired. Notably, the type of virus infection (persistent vs. acute) was a significant determinant in the expression of cytolytic molecules, suggesting a link between the prevalence of the target virus and the need for cytotoxicity for immune control. While CMV is known to induce terminal effector phenotypes in circulating T cells (Klenerman & Oxenius, 2016), studies have indicated that antigen persistence or virus titers alone do not fully explain cytolytic expression variations, as observed in HIV-specific CD8^+^ T cells across different disease severities (Sekine et al., 2020). Our data highlight the role of the tissue environment in modulating cytolytic molecule expression, revealing a complex hierarchy of molecular factors driving cytolytic molecule induction in tissues, pointing to a more intricate regulation of cellular cytotoxic machinery than previously recognized.

To better understand the molecular determinants for the expression of cytolytic molecules by mCD8^+^ T cells in tissues, we explored the tonsil CD8^+^ TRM cell response to IL-15 and TGF-β, key cytokines for TRM cells under both steady-state and inflammatory conditions in tissues (Mackay et al., 2015; Tieu et al., 2023). Despite low cytotoxic molecule expression *ex vivo*, mCD8^+^ T cells in tonsils acquired granzyme B and perforin, adopting an effector phenotype after overnight stimulation with IL-15, mirroring findings in human skin CD8^+^ TRM cells (Cheuk et al., 2017). These analyses suggest that high doses of IL-15 could override the effects of TGF-β and possibly other soluble and nonsoluble environmental factors, thereby inducing a cytotoxic profile of TRM cells in tissue locations. Nevertheless, this does not exclude the possibility of TGF-β exerting a dominant influence over IL-15 at steady-state doses, considering dose-dependent pleiotropic effects of TGF-β on immune cells (Taber et al., 2023). As key mediators of TRM differentiation and survival, IL-15 (Cui et al., 2014; Jabri & Abadie, 2015; Woon et al., 2016) and TGF-β (Hirai et al., 2021; Mani et al., 2019; Mohammed et al., 2016) are both abundantly expressed in various tissues and can often be found in overlapping locations amongst epidermal barrier regions. The coexistence of these cytokines, despite their opposing effects on TRM functions, raises the question of how they maintain homeostasis and effectively modulate effector cell fate during infection. The functional outcomes of these cytokines likely depend on the context and on different T cell subsets. Studies have underscored the significance of the context and temporal dynamics of these environmental cues – specifically, the sequential exposure to these signals is critical for the successful establishment of tissue residency (Pallett et al., 2017). Another explanation could be the extensive functional heterogeneity amongst the CD8^+^ TRM cell pool. Zitti et al have elegantly identified CD8^+^ TRMs with unique transcription factor profiles (Runx2) to represent subsets with pre-destined cytotoxic potential in human skin (Zitti et al., 2023). Such inherent heterogeneity could have an impact on their differential dependence and responsiveness to environmental stimuli. Taken together, these data suggest that mCD8^+^ T cell cytolytic effector properties in tissues are governed by a dynamic regulatory network. This network ensures that certain molecular thresholds can be surpassed in response to specific combinations of contextual triggers. Further fundamental exploration of this topic could shed light on the detailed functional kinetics and the overall state of viral immunity mediated by mCD8^+^ T cells across various tissues.

In our final set of experiments, we aimed to discern the functional and mechanistic significance behind the observed heterogeneity in granzyme expression among mCD8^+^ T cells in tonsils. Granzymes are typically categorized as cytolytic molecules, and single-cell RNA sequencing often labels them broadly as genes associated with cytotoxicity (Szabo et al., 2019). Our CRISPR-Cas9 KO experiments solidified the view that mCD8^+^ T cells in tissues depend heavily on GzmB and perforin for their cytolytic functions, hinting at more complex roles for the other granzymes in cell-mediated immunity beyond mere cell killing. Several nonclassical granzyme families have been implicated in noncytolytic functions, including the initiation of cytokine and inflammasome cascades under various stimuli across tissue and cell types (Anthony et al., 2010; Hildebrand et al., 2014; Zhou et al., 2020). In our experiments, a short-term culture of tonsil mCD8^+^ T cells with IL-15 and TGF-β had minimal effect on the expression of nonclassical serine proteases GzmA, GzmH, GzmK, and GzmM. Yet, when we extended the culture with TCR stimulation, such as with SEB to promote proliferation, GzmA, GzmH, and GzmM were upregulated in conjunction with cell division – a process tempered by TGF-β. These observations prompt further inquiry into the specific triggers and conditions necessary for the upregulation of individual granzymes and their subsequent effects on surrounding tissues. GzmK, however, stood out in our study for its unique expression profile. Unlike its counterparts, GzmK expression did not fluctuate with either short-term or extended stimulatory conditions, suggesting it operates under a distinctly different regulatory mechanism. This aligns with recent literature that positions GzmK-expressing T cells as a fundamental subset within tissue-associated T cells across various pathological states and tissues (Duquette et al., 2023; Jonsson et al., 2022). This distinctive expression pattern, particularly the higher presence in lymphoid tissues compared to peripheral blood, suggests that GzmK may play an essential role in tissue-specific immunity, possibly associated with the tissue remodeling and senescence observed in diseases such as cancer, autoimmune, and inflammatory conditions. Moreover, the role of GzmK in ‘inflammaging’ and its potential to modulate immune senescence, especially in fibroblasts of the elderly, highlights its significance in age-related inflammation (Arthur et al., 2021; Mogilenko et al., 2021). As such, GzmK emerges not just as a cytotoxic mediator but also as a molecule with diverse functions that may include tissue remodeling and regulatory roles within the immune system, warranting further exploration to fully understand its multifaceted contributions to tissue homeostasis and pathology.

In this study, we demonstrate the nuanced behaviors of mCD8^+^ T cells across various human tissues, uncovering the significant role of environmental cues and virus-specific interactions in determining the expression patterns of cytotoxic molecules. By analyzing human organ donors with paired tissue samples, we reveal that the cytotoxic activity of TRM cells is not uniform but rather is significantly influenced by the tissue of origin, the nature of the virus being targeted, and key regulatory cytokines like TGF-β and IL-15. Our findings indicate that the cytotoxic activity of human TRM cells is intricately regulated by environmental cues, which fine-tune their effector functions in both resting and activated states. This underscores the potential for manipulating TRM cells to enhance tissue-specific immunity, providing promising avenues for developing more effective treatments for infections and conditions associated with immune dysregulation.

## Materials and Methods

### Sample collection

Tissue samples from the spleen, liver, ileum, cecum, colon, MLNs, lung, LLNs, skin, and blood were collected from deceased human organ transplant donors at Karolinska University Hospital, Stockholm, and regional hospitals associated with the transplant division as part of the IHOPE tissue collection program. Tonsil samples and matched peripheral blood mononuclear cells were obtained from individuals undergoing tonsillectomy for recurrent infections or obstructive sleep apnea at ÖNH Odenplan, Stockholm, Sweden, as part of the TONCIM tissue collection program. Both studies were approved by the Regional Ethics Committee in Stockholm (Dnr 2019-05016 and 2021-03694, respectively), and written informed consent was obtained from all participants or their legal representatives during organ donation in accordance with the Declaration of Helsinki. Donor characteristics, including information on age, gender, and serology, are summarized in Table S1. The influence of gender was not specifically considered in the analysis of study data due to the limited sample size.

### Lymphocyte isolation from tissues

All tissues were processed in a digestion solution containing RPMI, 10% fetal bovine serum (FBS), 1% L-glutamine, 1% penicillin/streptomycin, and 25 μg/ml DNase I, as follows: Tissues from the spleen, LLNs, and MLNs were chopped into small pieces. These pieces were then ground through a cell strainer (pore size = 100 μm), filtered through another cell strainer (pore size = 70 μm), washed, and resuspended in the digestion medium. For the liver and lungs, tissues were chopped into small pieces and placed in 50 ml Falcon tubes. The tissue pieces were topped up with 50 ml of digestion medium and incubated at 37°C for 30 minutes on an orbital shaker at 120 rpm. After incubation, the pieces were ground through a cell strainer and washed as described above.

Gut components (ileum, cecum, colon) and skin were processed similarly, with the following modifications: For gut components, the digestion medium was supplemented with 80 μg/ml of purified collagenase (Worthington). Tissues were chopped into small pieces and placed in 50 ml Falcon tubes. The tissue pieces were topped up with 50 ml of digestion medium, ensuring they were never allowed to dry at any time. The tissues were then incubated at 37°C for 1 hour on an orbital shaker at 120 rpm. After incubation, the pieces were ground through a cell strainer and washed as above. Skin samples were processed by removing the subcutaneous fat layer with scissors and then isolating skin samples using 5 mm biopsy punches. The skin biopsy pieces were added to the digestion medium supplemented with approximately 240 μg/ml of purified collagenase. The tissues were then incubated at 37°C for 1 hour 30 minutes on an orbital shaker at 120 rpm. The pieces were ground through a cell strainer and washed as above. Cells were counted and cryopreserved in FBS containing 10% dimethyl sulfoxide (DMSO).

Tonsils were first cleaned of tonsil stones and blood clots and cut into small pieces. These pieces were then ground through a cell strainer (pore size = 100 μm), filtered through another cell strainer (pore size = 70 μm), washed, and resuspended in phosphate-buffered saline (PBS). Cells were cryopreserved in FBS containing 10% DMSO.

Peripheral blood mononuclear cells (PBMCs) from both cohorts were isolated from the blood via standard density gradient centrifugation and cryopreserved similarly. HLA typing for HLA-A2, -A3, -A24, -B7, and -B8 was performed using antibody staining and flow cytometry on whole blood.

### Intracellular cytokine staining

Flow cytometry staining was conducted on both fresh and cryopreserved cells. Initial phenotyping was performed on fresh cells, while subsequent functional and virus-specific analyses were carried out on cryopreserved cells. Cryopreserved cells were quickly thawed, resuspended in complete medium (RPMI 1640 containing 10% FBS, 1% L-glutamine, 1% penicillin/streptomycin) and supplemented with deoxyribonuclease I (DNase) (10 U/ml; Sigma-Aldrich). The cells were then rested at densities of 1-2 × 10^6 cells per well in 96-well U-bottom plates (Corning) at 37°C for 2 hours before use.

Cells were washed in PBS and stained with the LIVE/DEAD Aqua Dead Cell Stain Kit (Thermo Fisher Scientific) for 10 minutes at room temperature. Antibodies targeting chemokine receptors were added directly to the cell suspension with FACS buffer (PBS, 1% FBS, 2 mM EDTA) and incubated for 15 minutes at 37°C. This was followed by the addition of surface marker antibodies in FACS buffer and a further incubation of 30 minutes at room temperature. Cells were then washed in FACS buffer and fixed/permeabilized using the eBioscience™ FoxP3 Transcription Factor Staining Buffer Set (Thermo Fisher Scientific).

All subsequent staining and washing steps were performed using eBioscience™ 1X Permeabilization Buffer. Intracellular staining was conducted for 30 minutes at room temperature with fluorochrome-conjugated antibodies and biotinylated primary antibodies (anti-Granzyme H-biotin). After washing, cells were incubated with streptavidin-BV650 for 15 minutes at room temperature. Finally, the stained cells were washed, fixed in PBS containing 1% paraformaldehyde (PFA; Biotium), and acquired using a FACSymphony A5 (BD Biosciences). Flow cytometry reagents are detailed in Table S2.

### Analysis of public RNAseq dataset

Preprocessed and annotated single-cell RNA sequencing (scRNA-seq) data of T cells and innate lymphoid cells from Domínguez Conde et al. were downloaded in 2022 from the Tissue Immune Cell Atlas (https://www.tissueimmunecellatlas.org/#datasets). Only cells with the manual annotations TrmTemCD8, TrmGutCD8, and TemTemraCD8, and those originating from matching tissues in the IHOPE cohort (spleen, MLNs, lung, LLNs, liver, ileum, cecum, and blood) were selected for further downstream analysis. Cells from the colon were excluded as the data originated from only one donor.

### Functional assay

Cryopreserved tonsil cells or PBMCs were resuspended in complete medium (RPMI 1640 supplemented with 10% FBS, 1% L-glutamine, and 1% penicillin/streptomycin) at a concentration of 1 × 10^7 cells/ml and cultured at 1 × 10^6 cells/well in 96-well U-bottom plates (Corning). DMSO served as a negative control, while Staphylococcal Enterotoxin B (SEB) was used at a concentration of 0.5 μg/ml (Sigma) for stimulation. After 1 hour, brefeldin A (1 μl/ml; Sigma-Aldrich), monensin (0.7 μl/ml; BD Biosciences), and anti-CD107a-BV786 (clone H4-A3; Biolegend) were added. Cells were analyzed by flow cytometry following a 2, 3, 5, or 12-hour incubation at 37°C depending on the experiment.

### HLA class I tetramer generation and staining

HLA class I tetramers were generated as previously described (Price et al., 2005) using synthetic peptides (Peptides & Elephants GmbH. Biotinylated monomers were tetramerized by adding PE- or BV421-conjugated streptavidin at a 4:1 molar ratio. The specificities employed in this study included CMV A*0201 NV9 (NLVPMVATV), CMV A*2402 QL10 (QYDPVAALFL), CMV B*0702 TM10 (TPRVTGGGAM), CMV B*08:01 QV9 (QIKVRVDMV), EBV A*0201 GL9 (GLCTLVAML), EBV A*2402 TL9 (TYGPVFMCL), EBV B*07:02 RL9 (RPPIFIRRL), EBV B*0801 RL8 (RAKFKQLL), Flu A*0201 GL9 (GILGFVFTL), Flu B*07:02 LM9 (LPFDKTTVM), Flu B*08:01 EI9 (ELRSRYWAI), SARS-CoV-2 A*02:01 YL9 (YLQPRTFLL), SARS-CoV-2 A*02:01 VL9 (VLNDILSRL), SARS-CoV-2 A*02:01 TL9 (TLDSKTQSL), SARS-CoV-2 A*02:01 FV9 (FIAGLIAIV), SARS-CoV-2 A*02:01 RV9 (RLQSLQTYV), SARS-CoV-2 A*24:02 QI9 (QYIKWPWYI), SARS-CoV-2 A*24:02 NF9 (NYNYLYRLF), SARS-CoV-2 A*24:02 KF10 (KWPWYIWLGF), and SARS-CoV-2 B*07:02 SA9 (SPRRARSVA).

For tetramer staining, cryopreserved tissue cells were thawed, rested, and washed in PBS before being distributed at 5 × 10^6 cells per well in 96-well V-bottom plates. Cells were treated with dasatinib (50 nM; STEMCELL Technologies) for 15 minutes at room temperature prior to the addition of tetramers. After a further 20-minute incubation at room temperature, cells were washed in PBS. Staining for chemokine receptors, surface markers, and intracellular markers was then performed as previously described.

### Secretome quantification using proximity extension assay

Memory CD8^+^ T cells were magnetically enriched using the EasySep™ Human Memory CD8^+^ T Cell Enrichment Kit (STEMCELL Technologies) from blood and tonsils and stimulated with Immunocult™ Human CD3/CD28/CD2 T Cell Activator. Supernatants were collected after the indicated time points post-stimulation and stored at -80°C until analysis. Ninety-two proteins from the Immuno-Oncology Olink panel were assessed using the proximity extension assay technology (Olink AB).

### IL-15 and TGF-β treatment

Tonsil cells were thawed, stained with anti-CD69 BUV563 (BD Biosciences), resuspended in complete media at 2 × 10^6 cells/well in a 96-well U-bottom plate and rested for 2 hours at 37°C. Cells were then either left untreated or treated with IL-15 (20 ng/ml, PeproTech), TGF-β1 (20 ng/ml, R&D Systems), or both for 18 hours at 37°C before analysis by flow cytometry.

### Proliferation assay

Tonsil cells were labeled with Cell Trace Violet (2 mM; Thermo Fisher Scientific), resuspended in complete medium supplemented with normocin at 2 × 10^6 cells/mL, and cultured at 0.2 × 10^6 cells/well in 96-well U-bottom plates (Corning). When mentioned, cells were resuspended in complete medium supplemented with TGF-β1 (20 ng/ml; R&D Systems). Cells were either left unstimulated or stimulated with SEB (0.5 ug/ml; Sigma-Aldrich) and incubated for 4 days at 37°C before analysis by flow cytometry.

### Cas9 Ribonucleoprotein (RNP) Production

crRNA sequences for guide RNAs (gRNAs) were designed and tested. Individual or combinations of gRNAs targeting the same sequences that resulted in the highest editing efficiencies were selected. The corresponding sequences are listed in Table S3. A mixture of 80 µM tracrRNA and 80 µM crRNA (both from IDT DNA) was incubated at 95°C for 5 minutes, then allowed to cool to room temperature on the benchtop. Subsequently, 6 µM high-fidelity Cas9 (IDT DNA) was slowly added to the gRNA solution (tracrRNA + crRNA) to yield RNPs with 3 µM Cas9 and 20 µM gRNA, as well as 20 µM electroporation enhancer (IDT DNA). The RNPs were incubated for 15 minutes at RT and directly used for electroporation.

### CRISPR/Cas9-mediated knock-out

Tonsil cells were activated for two days in RPMI using Immunocult™ Human CD3/CD28/CD2 T Cell Activator (STEMCELL Technologies), 300 IU/ml recombinant human IL-2, 5 ng/ml recombinant human IL-7, and 5 ng/ml IL-15. Subsequently, 2 × 10^6 cells were electroporated (pulse code EH100) with 3.15 µl Cas9 RNP in 20 µl Nucleofector Solution P3 (Lonza, reference #V4SP 3096) using a 4D Nucleofector X unit (Lonza). After electroporation, cells were cultured in complete RPMI supplemented with 180 IU/ml IL-2 (PeproTech, #200-02) for two days before undergoing functional testing.

### Redirected cytotoxicity-killing assay

Tonsil cells (effector) were labeled with 1 µM CellTrace™ Violet, and P815 mastocytoma cells (TIB64, ATCC) (target) were labeled with 0.5 µM CellTrace™ Yellow for 10 minutes at 37°C in PBS, protected from light. After incubation, cells were thoroughly washed at least twice with complete medium. Tonsil cells were resuspended in complete medium, counted, and distributed in a V-bottom 96-well plate. P815 target cells, coated with anti-CD3 antibody (1 µg/ml; OKT3; Biolegend), were added to the effector cells to achieve effector-to-target (E:T) ratios of approximately 33:1, 11:1, 3.7:1, and 1.2:1. Cells were mixed thoroughly, then centrifuged briefly for 1 minute at 1100 rpm. After centrifugation, cells were incubated for 4 hours at 37°C. Following incubation, cells were treated with BD Pharmingen™ FITC Annexin V Apoptosis Detection Kit I according to the manufacturer’s instructions, with modifications including the addition of Live/Dead Aqua dye. Briefly, cells were washed in 1X binding buffer, then stained with LIVE/DEAD Aqua Dead Cell Stain in PBS for 30 minutes at room temperature in the dark. Cells were then thoroughly washed in PBS, followed by an additional wash with 1X binding buffer, and stained with Annexin V-FITC antibody in 1X binding buffer for 20 minutes at RT in the dark. After staining, cells were washed once in 1X binding buffer, resuspended in PBS, and analyzed by flow cytometry.

Cytolytic activity was quantified by subtracting the frequency of Annexin V^+^/Dead cell marker^+^ P815 cells in target-only wells (spontaneous lysis) from the frequency of Annexin V^+^/Dead cell marker^+^ P815 cells incubated with tonsil cells (effector-mediated lysis).

### Data analysis

Flow cytometry data were analyzed using FlowJo software version 10.7.1 (FlowJo LLC). The gating strategies are included in Fig. S1 and Fig. S2. Data was visualized and statistical analyses were performed using Prism version 10 for macOS (GraphPad Software Inc.). Differences between unmatched groups were analyzed using the Mann-Whitney test, while differences between matched groups were assessed using the Wilcoxon matched-pairs signed-rank test. Analyses between multiple groups were conducted with a Kruskal Wallis test with Dunn’s posttest for multiple comparisons. Correlations were evaluated using the Spearman rank correlation method. In some of the figures, data was analyzed and visualized using Python (v.3.8.8), pandas (v.1.2.4), scikit-learn (v.1.1.1), matplotlib (v.3.3.4), seaborn (v.0.11.1).

## Supplementary Material

Supplementary Material
