# Supplementary material for: Tissue origin and virus specificity shape human CD8+ T cell cytotoxicity
Source: Sci Immunol. Author manuscript; Available in PMC 2026 Oct 16. (PMC7619577; doi:10.1126/sciimmunol.adq4881)
Supplement: Supplementary file 1 [file EMS216125-supplement-Supplementary_Materials.pdf]

SUPPLEMENTARY FIGURES

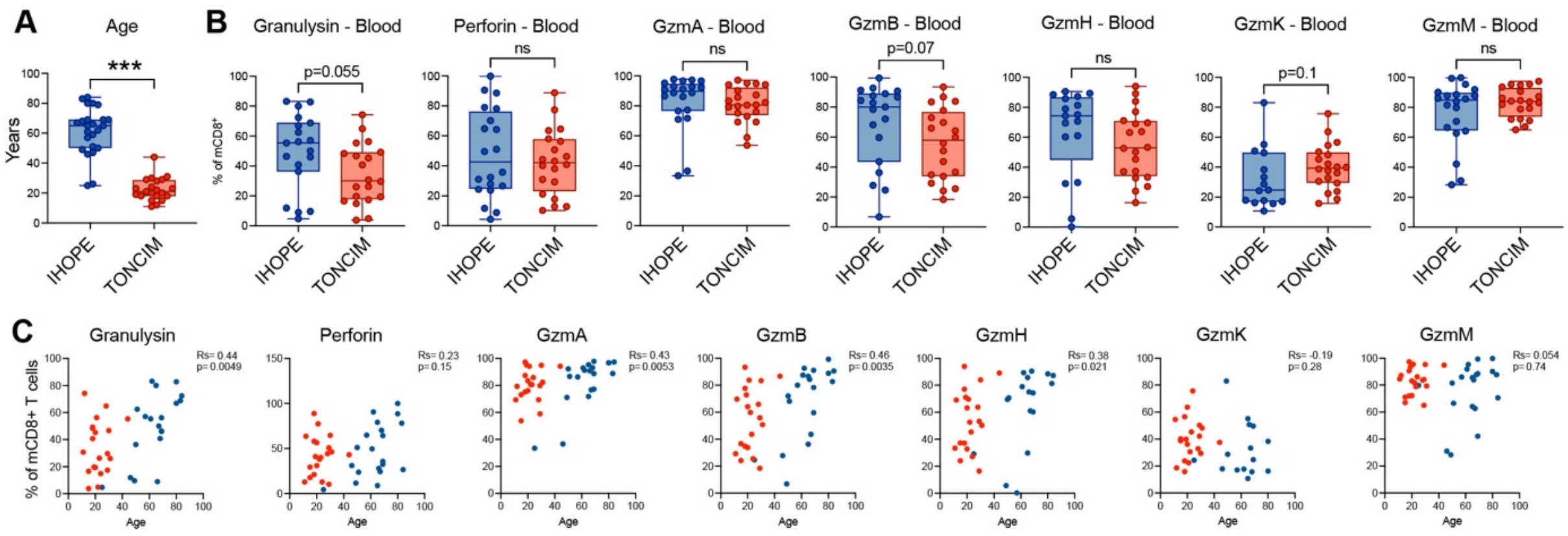

Suppl. Fig. 1. Cohort characteristics. Comparison of the IHOPE organ donor cohort versus the TONCIM tonsil donor cohort for **(A)** age and **(B)** cytotoxic molecule expression in blood mCD8+ T cells. **(C)** Correlations illustrating the relationship between age and cytotoxic molecule expression in blood mCD8+ T cells across the two cohorts.

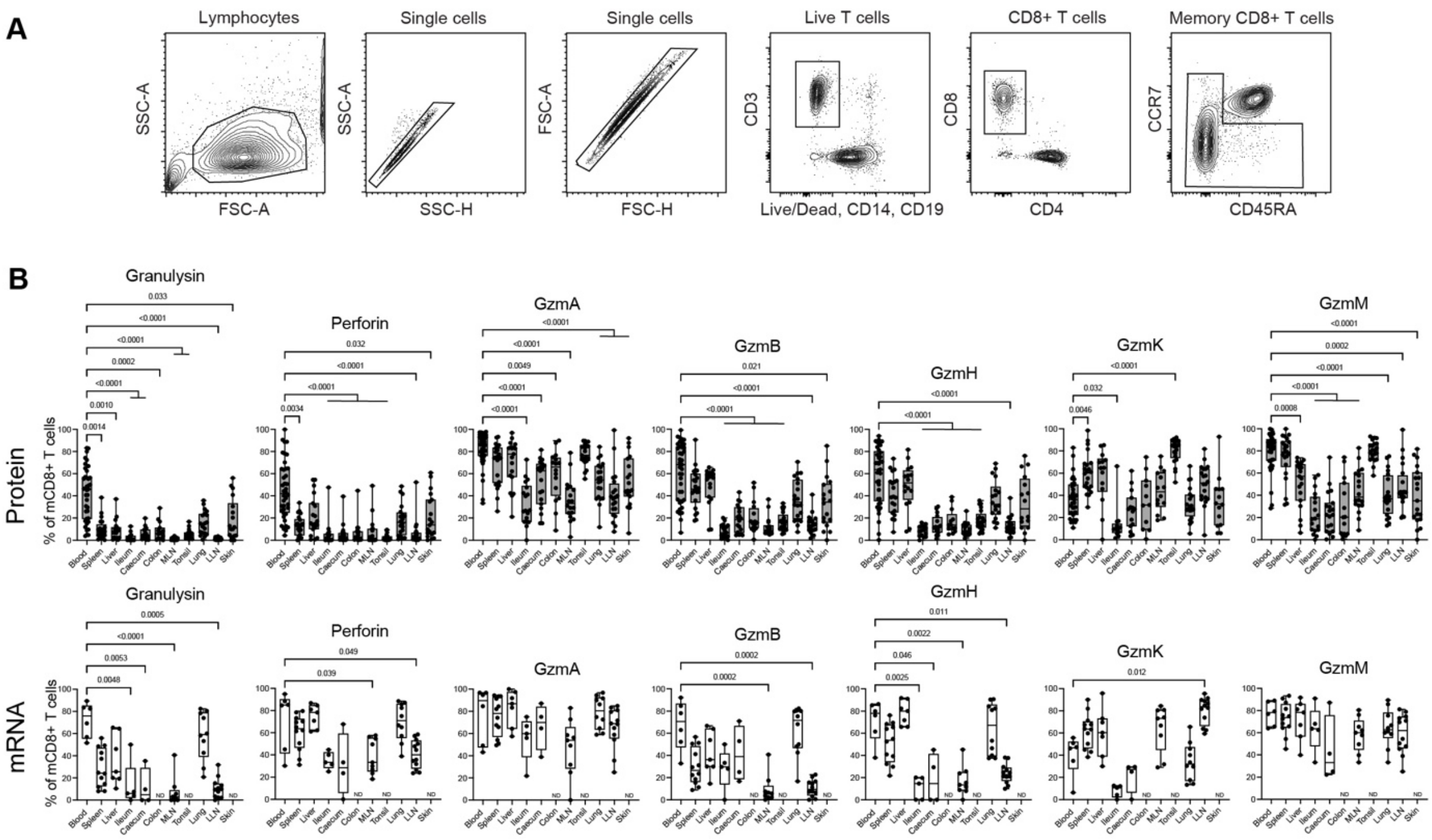

Suppl. Fig. 2. Gating strategies and comparative analysis of mCD8+ T cell cytotoxic molecule expression. (A) Gating strategy for mCD8+ T cells. **(B)** Comparison of relative cytotoxic molecule expression in mCD8+ T cells from indicated tissues at the protein level (in-house generated; top) and mRNA transcript level (public dataset; bottom).

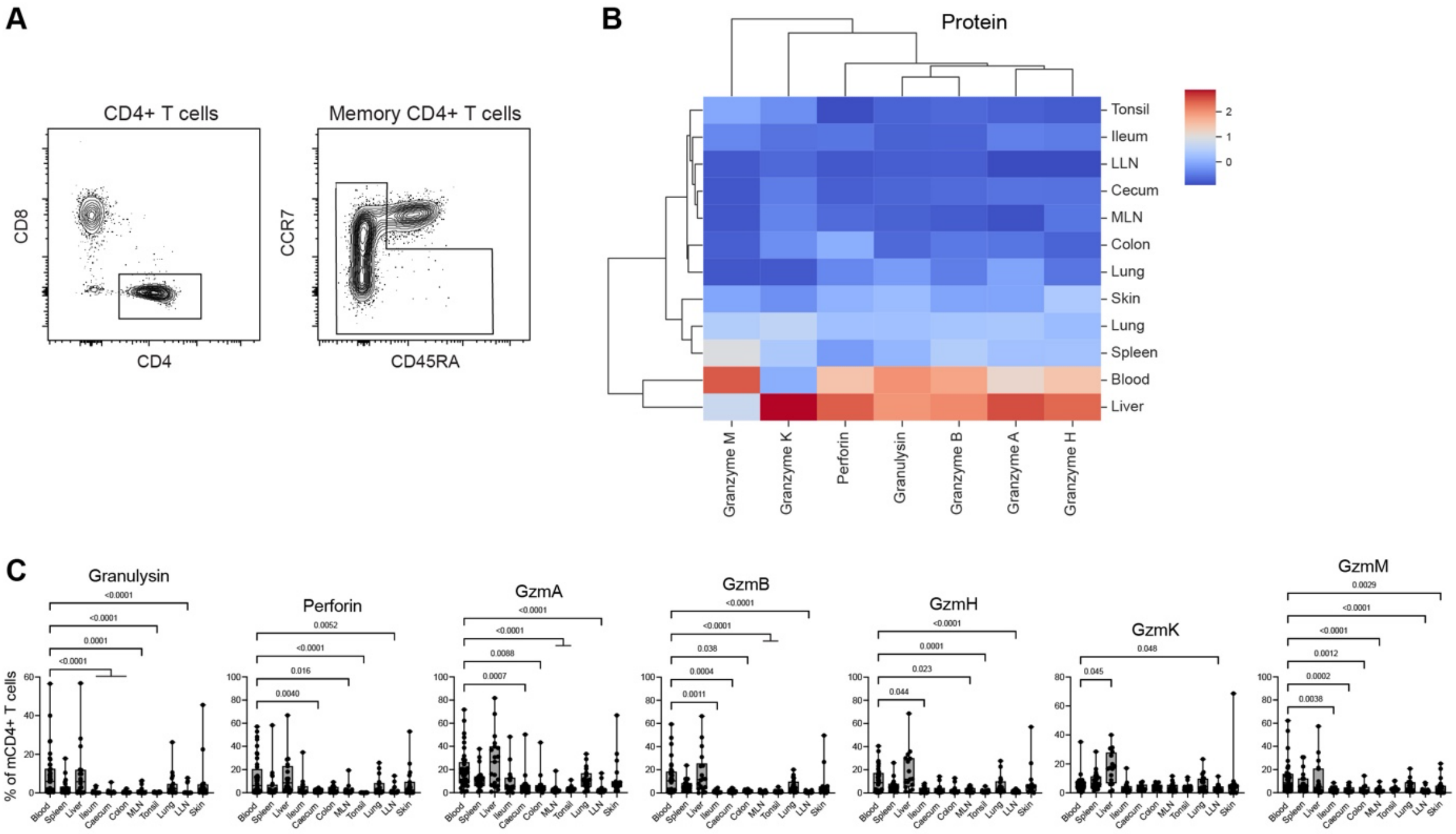

Suppl. Fig. 3. Cytotoxic molecule expression patterns in blood and vascularized tissues of mCD4+ T cells. (A) Gating strategy for mCD4+ T cells. **(B)** Heatmap illustrating cumulative data for cytotoxic molecule expression in mCD4+ T cells. **(C)** Relative cytotoxic molecule expression in mCD4+ T cells from indicated tissues at the protein level.

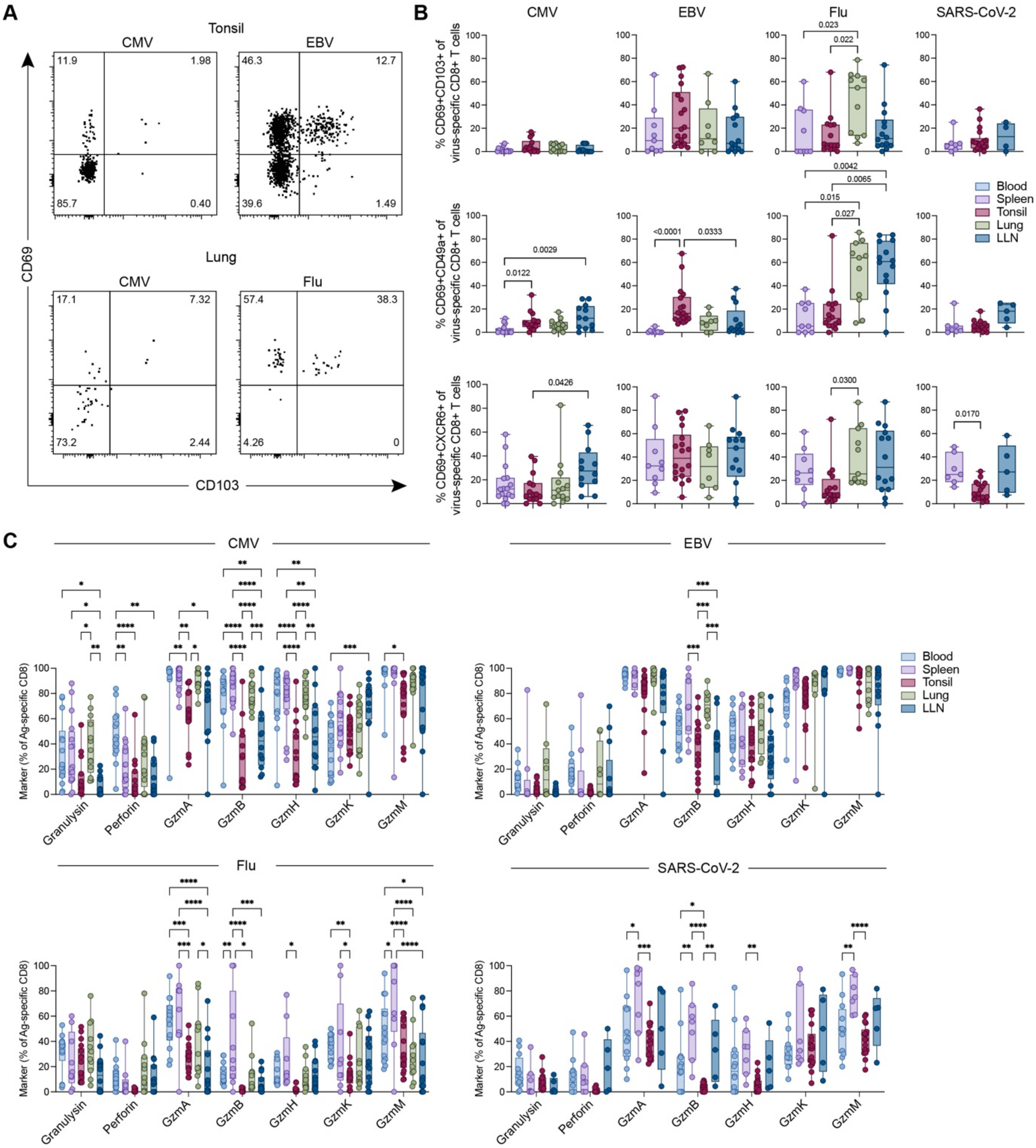

Suppl. Fig. 4. Virus-specific mCD8+ T cell cytotoxicity and tissue retention capabilities. (A) Representative flow cytometry plots showing CD69 and CD103 co-expression on virus-specific mCD8+ T cells from different tissues. **(B)** Frequencies of virus-specific mCD8+ T cells displaying indicated tissue residence markers and chemokine receptor combinations in different tissues. **(C)** Frequency analysis of virus-specific mCD8+ T cells expressing unique cytotoxic molecule combinations across different tissues.

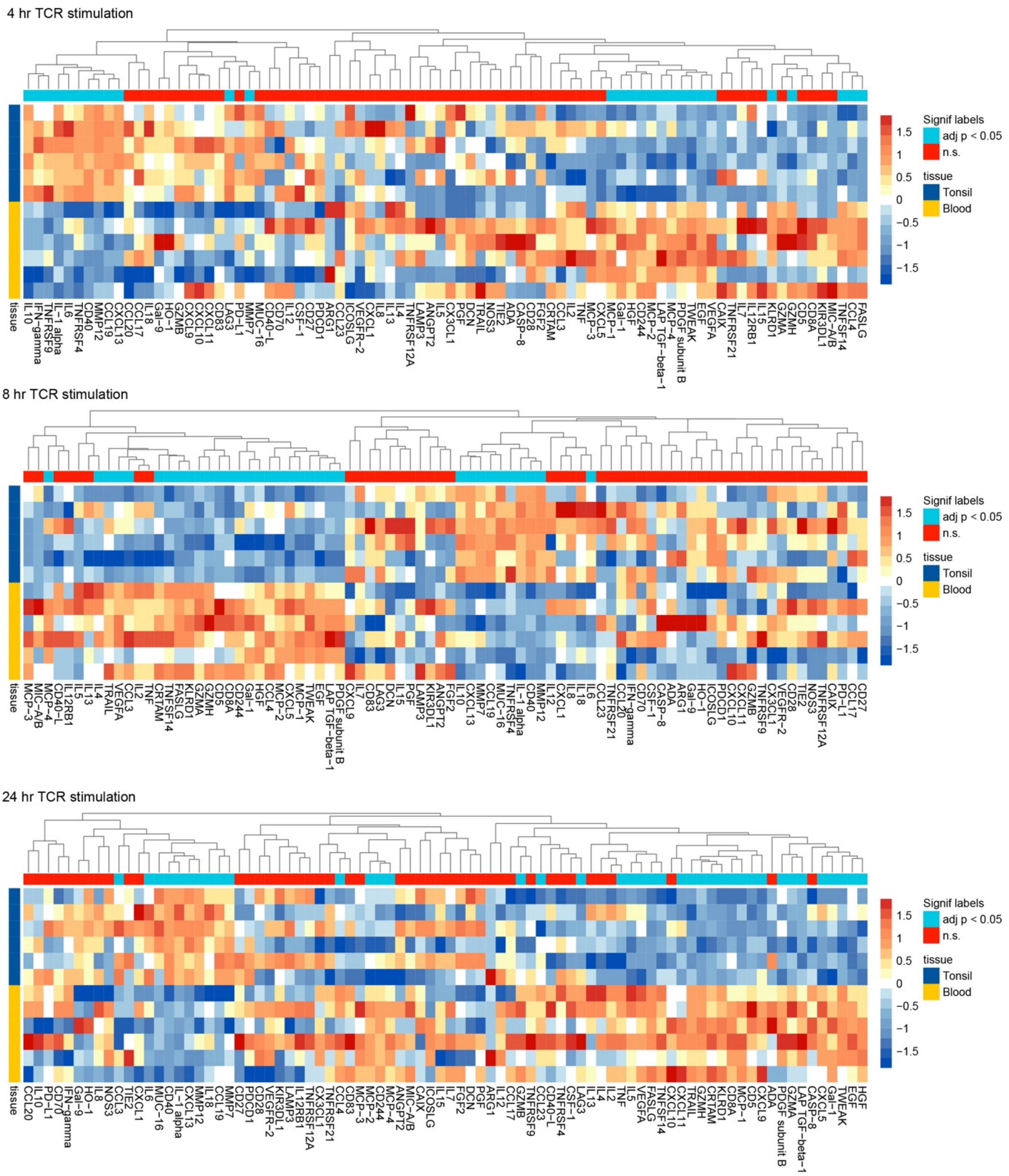

Suppl. Fig. 5. Secretome analysis of tonsil and blood mCD8+ T cells across different time points. Heat maps display the functional landscape of tonsil and blood mCD8+ T cells at 4 hours (top), 8 hours (middle), and 24 hours (bottom) following TCR stimulation.

SUPPLEMENTARY TABLES

Table S1: Donor characteristics for the organ donor (IHOPE) and tonsil (TONCIM) cohorts.

Donor	Age	Sex	Cohort	CMV status	EBV status	SARS-CoV-2 status
IHOPE1	25	M	IHOPE	positive	positive	ND
IHOPE2	67	M	IHOPE	positive	ND	ND
IHOPE3	51	F	IHOPE	positive	positive	ND
IHOPE4	83	F	IHOPE	positive	positive	ND
IHOPE5	61	M	IHOPE	positive	positive	ND
IHOPE6	69	M	IHOPE	positive	positive	ND
IHOPE7	84	M	IHOPE	positive	positive	ND
IHOPE8	62	F	IHOPE	positive	positive	ND
IHOPE9	66	M	IHOPE	positive	positive	ND
IHOPE10	47	M	IHOPE	positive	positive	ND
IHOPE11	65	M	IHOPE	positive	positive	ND
IHOPE12	68	M	IHOPE	positive	positive	ND
IHOPE13	26	M	IHOPE	positive	positive	ND
IHOPE14	80	M	IHOPE	positive	negative	ND
IHOPE15	65	M	IHOPE	positive	positive	ND
IHOPE17	79	M	IHOPE	negative	negative	ND
IHOPE18	69	M	IHOPE	positive	positive	ND
IHOPE20	46	M	IHOPE	negative	positive	ND
IHOPE21	59	M	IHOPE	positive	positive	ND
IHOPE22	69	F	IHOPE	positive	negative	ND
IHOPE24	50	M	IHOPE	positive	positive	ND
IHOPE25	49	M	IHOPE	positive	positive	ND
IHOPE26	57	M	IHOPE	positive	positive	ND
IHOPE27	80	M	IHOPE	positive	positive	ND
IHOPE28	62	M	IHOPE	positive	positive	ND
TONCIM11	21	M	TONCIM	ND	ND	Unexposed
TONCIM17	30	M	TONCIM	ND	ND	Infected
TONCIM18	19	F	TONCIM	ND	ND	Infected+Vaccinated

TONCIM21	24	F	TONCIM	ND	ND	Vaccinated
TONCIM29	23	F	TONCIM	ND	ND	Infected+Vaccinated
TONCIM30	20	F	TONCIM	ND	ND	Infected
TONCIM34	15	F	TONCIM	ND	ND	Infected
TONCIM42	12	F	TONCIM	ND	ND	Infected
TONCIM44	29	M	TONCIM	ND	ND	Vaccinated
TONCIM46	15	F	TONCIM	ND	ND	Infected+Vaccinated
TONCIM47	18	F	TONCIM	ND	ND	Infected+Vaccinated
TONCIM51	22	F	TONCIM	ND	ND	Infected+Vaccinated
TONCIM58	31	F	TONCIM	ND	ND	Infected
TONCIM59	29	F	TONCIM	ND	ND	Vaccinated
TONCIM68	20	F	TONCIM	ND	ND	Vaccinated
TONCIM74	44	M	TONCIM	ND	ND	Infected
TONCIM75	11	F	TONCIM	ND	ND	Infected
TONCIM103	18	F	TONCIM	ND	ND	Infected
TONCIM104	23	F	TONCIM	ND	ND	Infected
TONCIM122	28	M	TONCIM	ND	ND	Infected+Vaccinated
TONCIM140	18	M	TONCIM	ND	ND	Infected+Vaccinated

*ND = Not detected

Table S2. Flow cytometry reagents and analysis software.

	Reagent	Catalog number	Source	RRID
Antibodies	CCR7-APC-Cy7 (G043H7)	353212	Biolegend	AB_10916390
	CD103-BV605 (Ber-ACT8)	350218	Biolegend	AB_2564283
	CD107a-BV785 (H4A3)	328644	Biolegend	AB_2565967
	CD14-BV510 (M5E2)	301842	Biolegend	AB_2561946
	CD19-BV510 (HIB19)	302242	Biolegend	AB_2561668
	CD3-BUV805 (UCHT1)	612895	BD Biosciences	AB_2870183
	CD4-BUV496 (SK3)	612936	BD Biosciences	AB_2870220
	CD45RA-BV570 (HI100)	304132	Biolegend	AB_2563813
	CD49a-BUV615 (SR84)	751437	BD Biosciences	AB_2875433
	CD69-BUV563 (FN50)	748764	BD Biosciences	AB_2873167
	CD8-BUV395 (RPA-T8)	563795	BD Biosciences	AB_2722501
	CD95-BB630 (DX2)	624294	BD Biosciences	Custom
	CXCR3-PE-Cy5 (G025H7)	353756	Biolegend	AB_2904375
	CXCR5-PE-Vio615 (REA103)	130-123-912	Miltenyi Biotec	AB_2819538
	CXCR6-BUV661 (13B1E5)	749948	BD Biosciences	AB_2874182
	Granulysin-AF488 (RB1)	558254	BD Biosciences	AB_2869123
	Granzyme A-R718 (CB9)	566976	BD Biosciences	AB_2869982
	Granzyme B-BB790 (GB11)	624296	BD Biosciences	Custom
	Granzyme H-Biotin (polyclonal)	BAF1377	R&D	AB_2247969
	Granzyme K-PerCP-eFluor710 (G3H69)	46-8897-42	ThermoFisher	AB_2573854
	Granzyme M-eFluor660 (4B2G4)	50-9774-41	ThermoFisher	AB_2574373
	HLA-A2-PE-Cy7 (BB7.2)	343314	Biolegend	AB_2561571
	HLA-A24-FITC (220)	K0209-4	MBL	AB_592234
	HLA-A3-PE (GAP.A3)	566605	BD Biosciences	AB_2739760
	HLA-B7-APC (BB7.1)	372406	Biolegend	AB_2650776
	HLA-B8-APC-Vio770 (REA145)	130-099-583	Miltenyi Biotec	AB_2652004
	IFNg-PE (B27)	506507	Biolegend	AB_315440
	IL-2-PE-Dazzle594 (MQ1-17H12)	500344	Biolegend	AB_2564091
	PD-1-BUV737 (EH12.1)	612791	BD Biosciences	AB_2870118
	Perforin-BV421 (B-D48)	353307	Biolegend	AB_11149688

	Perforin-PE-Cy7 (B-D48)	353316	Biolegend	AB_2571973
	Streptavidin-BV650	405231	Biolegend	N/A
	TNFa-BV650 (MAb11)	563418	BD Biosciences	AB_2563884
Other reagents	BD GolgiStop	554724	BD Biosciences	AB_2869012
	Brefeldin A	420601	BioLegend	N/A
	Brilliant Stain Buffer Plus	566385	BD Biosciences	AB_2869761
	Dasatinib	73082	STEMCELL	N/A
	DNase I	4716728001	Sigma-Aldrich	N/A
	FoxP3 Transcription Factor Staining Buffer Set	00-5523-00	Thermo Fisher Scientific	N/A
	LIVE/DEAD Fixable Aqua Dead Cell Stain Kit	L34957	Thermo Fisher Scientific	N/A
	Paraformaldehyde	22023	Biotium	N/A
	Staphylococcal enterotoxin B (SEB)	S4881	Sigma-Aldrich	N/A
	FITC Annexin V Apoptosis Detection Kit I	556547	BD Pharmingen™	AB_2869082
Software	FlowJo	-	FlowJo, LLC	SCR_008520
	Prism	-	GraphPad Software Inc.	SCR_002798

Table S3. CRISPR-Cas9 guide RNAs

Target	crRNA 1	crRNA2	mode
TCR	AGAGTCTCTCAGCTGGTACA	GGAGAATGACGAGTGGACCC	in combination
Granzyme A	ACCATGTAGGGTCTTGAATG	AATCAAAGCCCCAGCACAGA	in combination
Granzyme B	TCCTTCAGGGGAGATCATCG	GATCATAAGATAAGCCATGT	in combination
Granzyme H	AACAAAGGCCATGTAGGGGC		single guide
Granzyme K	TTCAATATGGAAATTATTGG	AAGAAGTGTCACCTCATTCC	in combination
Granzyme M	CCCGATGATCTGGGTCCCAA	CCAGATCATCGGGGGCCGGG	in combination
Perforin	GAGGAGCAGACGGGCTGCCA	GATGCCCAGGAGGAGCAGAC	in combination
Control (PPP1R12C)	CCTCTAAGGTTTGCTTACGA		single guide
